# A super-enhancer-regulated RNA-binding protein cascade drives pancreatic cancer

**DOI:** 10.1038/s41467-023-40798-6

**Published:** 2023-09-06

**Authors:** Corina E. Antal, Tae Gyu Oh, Stefan Aigner, En-Ching Luo, Brian A. Yee, Tania Campos, Hervé Tiriac, Katherine L. Rothamel, Zhang Cheng, Henry Jiao, Allen Wang, Nasun Hah, Elizabeth Lenkiewicz, Jan C. Lumibao, Morgan L. Truitt, Gabriela Estepa, Ester Banayo, Senada Bashi, Edgar Esparza, Ruben M. Munoz, Jolene K. Diedrich, Nicole M. Sodir, Jasmine R. Mueller, Cory R. Fraser, Erkut Borazanci, David Propper, Daniel D. Von Hoff, Christopher Liddle, Ruth T. Yu, Annette R. Atkins, Haiyong Han, Andrew M. Lowy, Michael T. Barrett, Dannielle D. Engle, Gerard I. Evan, Gene W. Yeo, Michael Downes, Ronald M. Evans

**Affiliations:** 1 Gene Expression Laboratory, Salk Institute for Biological Studies, La Jolla, CA 92037 USA; 2 Moores Cancer Center, University of California San Diego, La Jolla, CA 92037 USA; 3 Department of Cellular and Molecular Medicine, University of California San Diego, La Jolla, CA 92093 USA; 4 The Francis Crick Institute, 1 Midland Rd, London, NW1 1AT UK; 5 Department of Surgery, Division of Surgical Oncology, University of California San Diego, La Jolla, CA 92037 USA; 6 Center for Epigenomics, University of California San Diego, La Jolla, CA 92037 USA; 7 Mayo Clinic in Arizona, Scottsdale, AZ 85259 USA; 8 Regulatory Biology Laboratory, Salk Institute for Biological Studies, La Jolla, CA 92037 USA; 9 Molecular Medicine Division, Translational Genomics Research Institute, Phoenix, AZ 85004 USA; 10 Mass Spectrometry Core for Proteomics and Metabolomics, Salk Institute for Biological Studies, La Jolla, CA 92037 USA; 11 HonorHealth Research Institute, Scottsdale, AZ 85258 USA; 12 Scottsdale Pathology Associates, Scottsdale, AZ 85260 USA; 13 Barts Cancer Institute, Queen Mary University of London, Charterhouse Square, London, EC1M 6BQ USA; 14 Storr Liver Centre, Westmead Institute for Medical Research and Sydney Medical School, University of Sydney, Westmead Hospital, Westmead, NSW 2145 Australia; 15 Sanford Stem Cell Institute, University of California San Diego, La Jolla, CA 92037 USA; 16 Present Address: Department of Pharmacology, University of California San Diego, La Jolla, CA 92093 USA; 17 Present Address: Department of Oncology Science, University of Oklahoma Health Sciences Center, Oklahoma City, OK 73117 USA; 18 Present Address: Genentech, Department of Translational Oncology, 1 DNA Way, South San Francisco, CA 94080 USA

**Keywords:** Pancreatic cancer, Epigenetics, Translation

## Abstract

Pancreatic ductal adenocarcinoma (PDAC) is a lethal malignancy in need of new therapeutic options. Using unbiased analyses of super-enhancers (SEs) as sentinels of core genes involved in cell-specific function, here we uncover a druggable SE-mediated RNA-binding protein (RBP) cascade that supports PDAC growth through enhanced mRNA translation. This cascade is driven by a SE associated with the RBP heterogeneous nuclear ribonucleoprotein F, which stabilizes protein arginine methyltransferase 1 (PRMT1) to, in turn, control the translational mediator ubiquitin-associated protein 2-like. All three of these genes and the regulatory SE are essential for PDAC growth and coordinately regulated by the Myc oncogene. In line with this, modulation of the RBP network by PRMT1 inhibition reveals a unique vulnerability in Myc-high PDAC patient organoids and markedly reduces tumor growth in male mice. Our study highlights a functional link between epigenetic regulation and mRNA translation and identifies components that comprise unexpected therapeutic targets for PDAC.

## Abstract (web summary)

The epigenetic mechanisms underlying pancreatic ductal adenocarcinoma (PDAC) are not fully elucidated. Here, the authors reveal a druggable super-enhancer-mediated RNA-binding protein cascade that supports PDAC growth through enhanced mRNA translation.

## Introduction

With a 5-year survival rate of 11%, pancreatic cancer is predicted to become the second leading cause of cancer-related death in the U.S. this decade, with pancreatic ductal adenocarcinoma (PDAC) accounting for >90% of all pancreatic cancer cases^1,2^. While the common mutational landscape and drivers of PDAC have been identified, this knowledge has yet to translate into durable treatments, as targets such as Kras and Myc have proven intractable^3,4^. Consequently, identifying vulnerabilities in molecular pathways required to sustain tumor growth is an area of active research.

RNA-binding proteins (RBPs) post-transcriptionally regulate RNA splicing, stability, polyadenylation, localization, and translation. As such, RBPs have been implicated in all aspects of cancer development and progression, and their aberrant expression correlates with decreased survival^5^. Consequently, RBPs have emerged as a class of potential cancer therapeutic targets, particularly in Myc-driven tumors^6–9^. To sustain the transformed cancer phenotype, tumor cells leverage multiple aspects of post-transcriptional gene regulation, including increased mRNA translation, with Myc as a master regulator^10–13^. In PDAC, protein synthesis is elevated both in vitro and in vivo in tumor cells compared to normal tissue^14,15^. These findings suggest a dependency on increased translation in PDAC cells that may be therapeutically targeted, as PDAC is more sensitive to protein synthesis inhibition than normal tissue^10,14^. There are a number of translation initiation inhibitors targeting the mammalian target of rapamycin complexes that are currently under clinical investigation in various cancers, and omacetaxine, an inhibitor of protein elongation, is approved by the FDA for the treatment of chronic myeloid leukemia^16,17^. However, alternative therapeutic avenues targeting translation are needed as resistance, compensatory mechanisms, and toxicity have hindered the success of previous strategies^17^. Targeting ribosome biogenesis, particularly in the context of Myc hyperactivation, has only recently been appreciated as a promising avenue^18^. This highly regulated process is closely linked to cellular proliferation^19^ as cancer cells upregulate ribosome biogenesis and are thus highly sensitive to drugs inhibiting rRNA transcription or maturation.

Super-enhancers (SEs) are genomic regions with high transcription factor binding densities and active histone marks that function as regulatory nodes at the transcriptional level to establish cell identity and behavior^20^. SEs are key drivers of tumorigenesis, having been identified at oncogenes and other genes that comprise regulatory clades overseeing the transformation and proliferation of cancer cells^20,21^. While several SE-driven transcription factors have been implicated in PDAC cell identity^22^, those coordinating the sustained increase in translation required for proliferation remain largely unexplored. Given that 95% of PDAC is driven by oncogenic KRAS mutations^23^, and that Ras-driven tumors are dependent on ribosomal biogenesis and translation control^24^, intercepting oncogene addiction by inhibiting protein synthesis has therapeutic potential in PDAC. However, druggable nodes in this pathway remain limited^23^.

Here, we uncover a Myc-coordinated SE-regulated network that upregulates translation through increased ribosome biogenesis in order to sustain the transformed cancer phenotype. This cascade is comprised of the post-transcriptional effector heterogeneous nuclear ribonucleoprotein F (hnRNP F) that mediates the stability of protein arginine methyltransferase 1 (Prmt1), which in turn modifies ubiquitin-associated protein 2-like (Ubap2l), an RBP that directly regulates translation, to affect its RNA-binding ability. Importantly, intercepting this interlinked SE cascade at any of the downstream nodes is sufficient to suppress protein translation and inhibit cancer progression. These data provide a link between the functional consequence of a SE in the nucleus and ribosome biogenesis in the cytoplasm that can be targeted as a vulnerability in PDAC, and potentially other Myc-driven cancers.

## Results

### Super-enhancer mapping identifies a critical role for *HNRNPF* in tumor growth

**Fig. 1:**
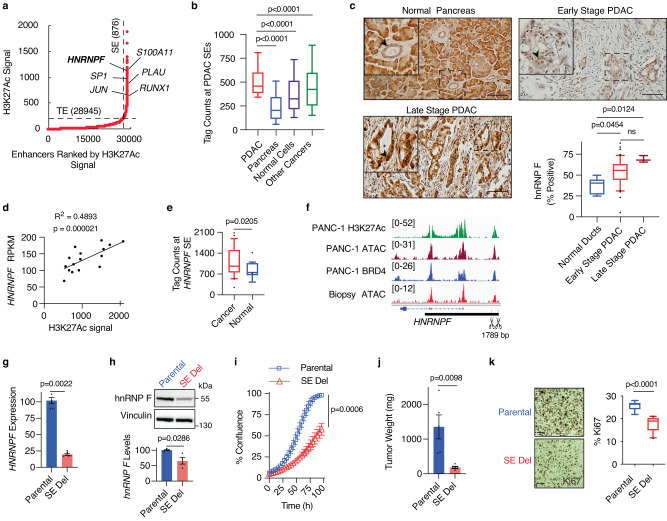
Perturbation of the *HNRNPF* super-enhancer impairs tumor growth. **a** Super-enhancers (SEs) and typical enhancers (TE) plotted based on their input-normalized H3K27Ac ChIP-seq signal. **b** Box plots showing cumulative tag counts across all identified SE loci (*n* = 876) in 16 PDAC cell lines, 2 normal pancreas tissue samples, 8 normal cell types (293T, NHEK, myoblasts, monocytes, bronchial epithelial cells, skeletal myotubes, keratinocytes, and macrophages), and 6 other cancer cell lines (breast, colon, liver, lung, cervical, and blood cancers). **c** Representative images and quantification of hnRNP F IHC from a human PDAC tissue microarray containing normal pancreas (*n* = 5), early-stage (*n* = 70), or late-stage (*n* = 3) PDAC. Arrows point to ductal cells in the normal pancreas and to tumor cells in PDAC. **d** Scatter plot of *HNRNPF* expression and H3K27Ac signal at the *HNRNPF* SE in 16 human PDAC cell lines. **e** Box plots showing cumulative tag counts at the *HNRNPF* SE in all cancer cell lines listed above (*n* = 23) compared to normal tissues (*n* = 10; pancreas tissues and normal cell lines listed above). **f** Genome browser tracks showing H3K27Ac signal, open chromatin regions, and BRD4 signal at the *HNRNPF* locus. The black bar indicates the SE, and scissors indicate the deleted base pairs in the SE deleted cells. **g** RT-qPCR showing *HNRNPF* expression, normalized to *GAPDH*, in MIA PaCa-2 parental and *HNRNPF* SE deleted cells (*n* = 6 biological replicates). **h** Representative immunoblot (top) and quantification (bottom) showing hnRNP F protein levels in MIA PaCa-2 parental and *HNRNPF* SE deleted cells (*n* = 4 biological replicates). **i** Cell confluence determined using IncuCyte software from phase-contrast images of MIA PaCa-2 parental and *HNRNPF* SE deleted cells (*n* = 3 independent experiments). **j** Tumor weights from mice orthotopically transplanted with MIA PaCa-2 parental (*n* = 5) or *HNRNPF* SE deleted (*n* = 5) cells. **k** Representative images (left;) and quantification (right) of tumor sections stained with Ki67 from 2 fields per biological replicate (*n* = 10). Box plots indicate median (middle line), 25th, 75th percentile (box), 10th and 90th percentile (whiskers), and outliers (single points) for (**c**), (**e**) and (**k**), with outliers omitted for (**b**). Data represent means ± SEM in (**g**), (**h**), and (**j**) and means ± SD in (**i**). Two-sided Friedman test with Dunn’s multiple comparisons for matched non-parametric data was used in (**b**), unpaired Mann–Whitney test in (**g**, **h**), and unpaired two-tailed t-test in (**i**–**k**). ns: not significant. Scale bar: 100 μm. Source data are provided as a Source Data file.

To identify SEs driving PDAC cell identity and pathogenesis, we mapped the genomic locations of SEs in 16 human pancreatic cancer cell lines, both primary and established. Using H3K27ac as a preferred SE identifier^20^, we delineated 876 SEs (Fig. 1a) among these biological replicates. Genes juxtaposed to these SEs encode proteins previously implicated in processes dysregulated in cancer, including cell proliferation (JUN^25^, S100A11^26^, and PLAU^27^) and transcription (SP1^28^ and RUNX1^29^). Consistent with the role these SEs play in PDAC, the H3K27Ac signal at these same loci is lower in a panel of normal pancreas tissues or normal cell lines (Fig. 1b). However, the signal at these SEs in other cancers, including breast, colon, liver, lung, cervical, and blood cancers, was varied, exhibiting some commonalities to certain cancers but not others. Moreover, transcription factor motif analysis of the nucleosome-free regions contained within the identified SE peaks revealed activator protein-1 (AP-1) complex members, including JUN, FOS, and ATF family members, as the top nine enriched motifs. This is consistent with previous work identifying AP-1 transcription factors as downstream mediators of mutant KRAS in PDAC^30^.

Surprisingly, we identified *HNRNPF*, a regulator of alternative splicing, polyadenylation, and RNA stability^31,32^, among the top 25 SE-associated genes. Immunohistochemical evaluation of hnRNP F protein expression in clinically annotated human PDAC samples revealed that hnRNP F is upregulated in the epithelial tumor compartment of early and late-stage PDAC compared to normal ducts (Fig. 1c). Corroborating these results, analysis of publicly available human single cell (sc)RNA-seq data^33,34^ showed that *HNRNPF* is also upregulated at the RNA level in PDAC cells compared to normal ducts and that its expression increases with tumor stage (Supplementary Fig. 1a, b). Supporting the role of this SE in mediating *HNRNPF* expression, we found a significant correlation between the H3K27Ac signal at this SE and expression of the *HNNRPF* gene in the 16 PDAC cell lines we assessed (Fig. 1d). Indeed, given this correlation and the fact that *HNRNPF* has been shown to be upregulated in other cancers^35–37^, we find that the H3K27Ac signal at the *HNRNPF* SE is higher in cancer cells compared to normal cells and tissues (Fig. 1e). Further supporting the functionality of this SE, increased chromatin accessibility was found at sites of H3K27ac at the *HNRNPF* locus in PDAC cell lines as well as in aneuploid epithelial tumor cell nuclei obtained from a PDAC patient biopsy (Fig. 1f and Supplementary Fig. 1c–e) using our previously established method^38^, supporting the relevance of SE-regulated *HNRNPF* expression in human PDAC. Since BRD4 is a transcriptional coactivator enriched at SEs, we also analyzed publicly available BRD4 ChIP-seq data from PANC-1 cells and identified a SE associated with *HNRNPF* among the top 20 SE-associated genes (Fig. 1f and Supplementary Fig. 1f). Based on these findings, we hypothesized that SE regulation of *HNRNPF* is a key driver of PDAC growth and that it likely plays a role in other cancers.

To establish a functional role for this SE in driving *HNRNPF* expression and consequently tumor growth, we deleted ~1800 bases spanning the 5′ distal enhancer in the human MIA PaCa-2 PDAC cell line (Fig. 1f; Supplementary Fig. 1g). Deletion of the SE element resulted in an 80% reduction in *HNRNPF* transcript levels (Fig. 1g), leading to a 35% reduction in protein levels (Fig. 1h). Surprisingly, homozygous deletion of this distal putative regulatory region led to a reduction in chromatin accessibility at another peak that is part of the *HNRNPF* SE (Supplementary Fig. 1h), suggesting these chromatin regions potentially interact to regulate *HNRNPF* expression. Our analysis did not reveal any additional changes in chromatin accessibility ±1 Mb of the *HNRNPF* SE, which is where enhancers are typically found^39^, or even up to 3 Mbs up- or downstream of the *HNRNPF* locus. Functionally, the *HNRNPF* SE deleted cells were less proliferative in 2-dimensional (2D) cultures, which culminated in a 42% reduction in cell confluence after 100 h (Fig. 1i) and established smaller colonies in a soft agar 3D culture assay (Supplementary Fig. 1i). Similar results were observed for *HNRNPF* mRNA and protein levels, as well as 2D proliferation rates, upon deletion of the same SE region in a separate human PDAC cell line, PANC-1 (Supplementary Fig. 1j–m). Importantly, re-expression of *HNRNPF* in these SE deleted cells partially rescued the proliferative defect. Cumulatively, these data demonstrate that *HNRNPF* is the predominant gene driven by this SE that is responsible for driving proliferation in PDAC cells.

Most strikingly, orthotopic transplantation of the MIA PaCa-2 SE deleted cells into the pancreas of immunodeficient mice generated tumors that were 85% smaller by weight (4 weeks post-transplantation) than those induced by the MIA PaCa-2 parental cells (Fig. 1j). This decrease in tumor burden corresponded with a significant decrease in in vivo proliferation, as evidenced by a 30% reduction in Ki67 staining in the SE deletion-derived tumors (Fig. 1k). In combination, these findings support a functional role for SE-regulated *HNRNPF* expression in vivo, in tumor growth.

### Perturbation of the *HNRNPF* gene impairs tumor growth

**Fig. 2:**
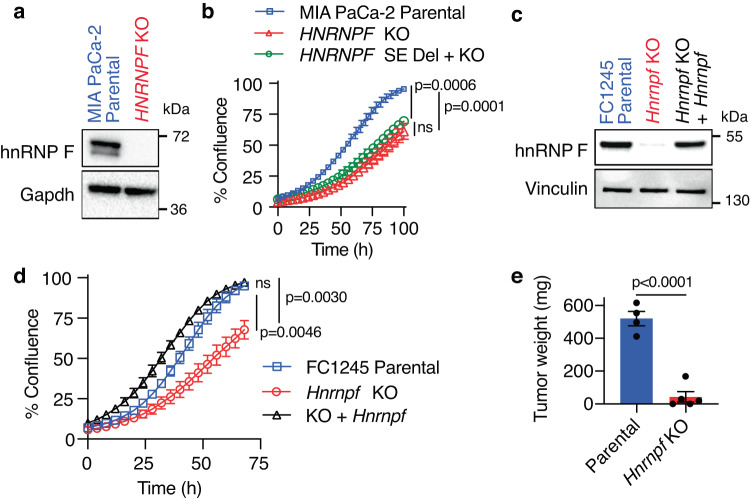
Perturbation of the *HNRNPF* gene impairs tumor growth. **a** Representative immunoblot from two independent experiments showing hnRNP F protein levels in MIA PaCa-2 parental or *HNRNPF* KO cells. **b** Cell confluence determined using IncuCyte software from phase-contrast images of MIA PaCa-2 parental cells, *HNRNPF* KO cells, or *HNRNPF* SE deleted cells in which the *HNRNPF* gene was also knocked out (*n* = 3 independent experiments). **c** Representative immunoblot from two independent experiments showing hnRNP F levels in FC1245 parental, *Hnrnpf* KO, or *Hnrnpf* KO cells rescued with re-expression of exogenous *HNRNPF*. **d** Cell confluence determined using IncuCyte software from phase-contrast images of FC1245 parental, *Hnrnpf* KO, or *Hnrnpf* KO cells rescued with re-expression of exogenous *HNRNPF* (*n* = 3 independent experiments). **e** Tumor weights from mice orthotopically transplanted with FC1245 parental (*n* = 4) or *Hnrnpf* KO (*n* = 5) cells. Data represent means ± SEM in (**d**) and (**e**) and means ± SD in (**b**). One-way ANOVA with Sidak’s multiple comparisons test was performed in (**b**) and (**d**) and unpaired two-tailed t-test in (**e**). ns: not significant. Source data are provided as a Source Data file.

To causally associate the reduced tumor burden seen following deletion of the SE element with altered *HNRNPF* expression, CRISPR/Cas9-mediated gene editing was used to knockout (KO) *HNRNPF* in MIA PaCa-2 cells (Fig. 2a). Transcriptomic analysis revealed that 62% of genes downregulated upon *HNRNPF* KO are accounted for by deletion of the SE and that there were no gene expression changes within 3 Mb of the *HNRNPF* SE that were uniquely present in the SE deleted cells but absent from KO cells (Supplementary Fig. 2a). Similar to deletion of the SE, KO of *HNRNPF* also reduced cell proliferation, but deletion of the SE in the KO cells did not lead to further suppression of proliferation (Fig. 2b). These data suggest that deletion of the SE does not have hnRNP F-independent off-target effects that further impact proliferation and that there could be a threshold effect, with small changes in hnRNP F expression disproportionately affecting its RNA targets. Moreover, these findings highlight the importance of SE in regulating *HNRNPF* levels in cancer.

**Fig. 3:**
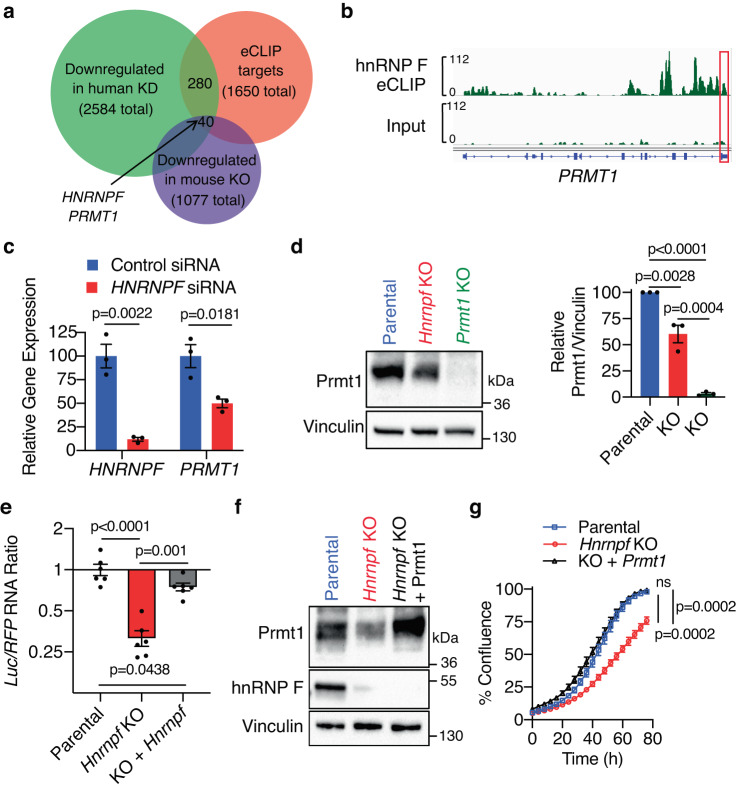
hnRNP F regulates PRMT1 levels to control cellular proliferation. **a** Venn diagram showing the overlap among eCLIP targets in AA0779E cells, genes downregulated upon *HNRNPF* knockdown in AA0779E cells, and genes downregulated upon *Hnrnpf* KO in mouse FC1245 cells. **b** Browser tracks displaying hnRNP F binding to *PRMT1* mRNA; 3’UTR is boxed in red. **c** RT-qPCR showing expression levels of *HNRNPF* and *PRMT1*, normalized to *GAPDH*, in MIA PaCa-2 cells knocked down with siRNA against *HNRNPF* or a non-targeting control (*n* = 3 biological replicates). **d** Representative immunoblot (left) and quantification (right) showing Prmt1 levels in FC1245 parental and *Hnrnpf* KO cells (*n* = 3 biological replicates). **e** RT-qPCR showing levels of an exogenously expressed luciferase gene containing the 3’UTR of *Prmt1*, normalized to RFP driven by an independent promoter on the same plasmid, in FC1245 parental, *Hnrnpf* KO, or *Hnrnpf* KO cells rescued by exogenous *HNRNPF* re-expression (*n* = 6 biological replicates). **f** Representative immunoblot from two independent experiments showing Prmt1 and hnRNP F levels in FC1245 parental, *Hnrnpf* KO, or *Hnrnpf* KO cells rescued with re-expression of exogenous Prmt1. **g** Cell confluence determined using IncuCyte software from phase-contrast images of FC1245 parental, *Hnrnpf* KO, or *Hnrnpf* KO cells rescued with re-expression of exogenous Prmt1 (*n* = 3 independent experiments). Data represent means ± SEM in (**c**–**e**) and (**g**). Unpaired two-tailed t-test with two-stage step-up multiple comparison correction was performed in (**c**), one-way ANOVA followed by Tukey’s multiple comparisons test in (**d**, **e**), and one-way ANOVA with Sidak’s multiple comparisons test in (**g**). ns: not significant. Source data are provided as a Source Data file.

In order to assess the in vivo effect of knocking out *HNRNPF* in immune-competent mice, we next knocked out *Hnrnpf* in the *Kras*^*LSL-G12D/+*^*;Trp53*^*LSL-R172H/+*^*;Pdx-1-Cre* (KPC)-derived cell line, FC1245 (Fig. 2c). KO of *Hnrnpf* significantly compromised proliferation in both 2D (Fig. 2d) and 3D (Supplementary Fig. 2b) cultures, largely replicating the phenotypes observed with deletion of the SE element or deletion of the gene in human PDAC cells. Moreover, this proliferation defect was fully rescued by restoring *Hnrnpf* expression in the KO clone to a level similar to the parental cells (Fig. 2c, d). Comparable to deleting the *HNRNPF* SE element, KO of *Hnrnpf* led to a 90% reduction in tumor burden in orthotopically transplanted syngeneic mice (Fig. 2e), further substantiating the role of *Hnrnpf* in tumor growth.

### hnRNP F regulates *PRMT1* expression to control cellular proliferation

To determine how hnRNP F regulates tumor cell growth, its interacting RNAs were identified in the primary human PDAC cell line AA0779E using enhanced cross-linking and immunoprecipitation (eCLIP) followed by high-throughput sequencing^40^. Gene ontology analysis of the >1600 bound RNAs (Supplementary Data 1) revealed an enrichment of transcripts involved in chromatin modifications, cell signaling, and cell cycle (Supplementary Fig. 3a). Overlapping these targets with those whose expression was reduced upon *Hnrnpf* knockdown in the same cell line identified 320 RNAs (~20% of its binding targets) that are dependent on hnRNP F binding to maintain transcript levels (Fig. 3a). Of these targets, 40 were similarly affected upon *Hnrnpf* knockout in the mouse FC1245 cell line. Ontology analysis of this subset of 40 evolutionarily conserved targets revealed enrichment of multiple aspects of protein translation (Supplementary Fig. 3b). Among its targets were *HNRNPF*, in agreement with its auto-regulation^31^, and protein arginine methyltransferase 1 (PRMT1) (Fig. 3a, b), which was recently shown to regulate translation initiation^41^. As the enzyme responsible for the majority of asymmetric arginine dimethylation^42^, PRMT1 is elevated in a multitude of cancers, including PDAC, where its expression positively correlates with tumor size and clinical outcome^43–45^. A small molecule inhibitor of type I PRMTs, which PRMT1 belongs to, is currently in clinical trials for solid tumors and diffuse large B-cell lymphoma^46^. In PDAC patients, *PRMT1* expression is increased ~3 fold compared to normal pancreas (Supplementary Fig. 3c). Corroborating these results, using human scRNA-seq data^33,34^, we show that *PRMT1* is also upregulated in PDAC cells compared to normal ducts (Supplementary Fig. 3d) and that its expression increases with tumor stage (Supplementary Fig. 3e); however, how it contributes to tumor progression is not fully understood.

Supporting *PRMT1* as a downstream target, the transient knockdown of *HNRNPF* in MIA PaCa-2 cells reduced *PRMT1* mRNA expression by 50% (Fig. 3c). Corroborating these results across species, knockout of *Hnrnpf* in mouse KPC cells led to a 30% reduction in *Prmt1* mRNA (Supplementary Fig. 3f), which resulted in a 40% reduction in protein levels (Fig. 3d). Since *Hnrnpf* KO had no effect on *Prmt1* splicing (Supplementary Fig. 3g), we investigated if hnRNP F is required to stabilize *PRMT1* transcripts. To do so, we utilized a mRNA stability assay in which the human *PRMT1* 3’ UTR containing the hnRNP F binding sites was cloned downstream of a luciferase cassette in a reporter that included RFP for normalization. Compared to the parental line, luciferase expression was decreased by 70% in *Hnrnpf* KO cells upon transient transfection of the reporter (Fig. 3e). This effect was largely reversed by exogenous *Hnrnpf* expression. In line with these results, *Prmt1* mRNA has a shorter half-life in the absence of *Hnrnpf* as observed upon inhibition of transcription (Supplementary Fig. 3h). Given that these findings support the regulation of *Prmt1* transcripts by hnRNP F, we assessed whether restoring *Prmt1* expression in the *Hnrnpf* KO cells is sufficient to rescue the proliferative defect. Indeed, exogenously expressing wild-type *Prmt1* transcript variant 1 to a level similar to the parental line, but not a catalytically dead version, rescued the proliferation defect of *Hnrnpf* KO cells (Fig. 3f, g and Supplementary Fig. 3i–k). This demonstrates that Prmt1 is a major downstream target of hnRNP F that modules cell proliferation.

### Loss of Prmt1 impedes tumor growth

**Fig. 4:**
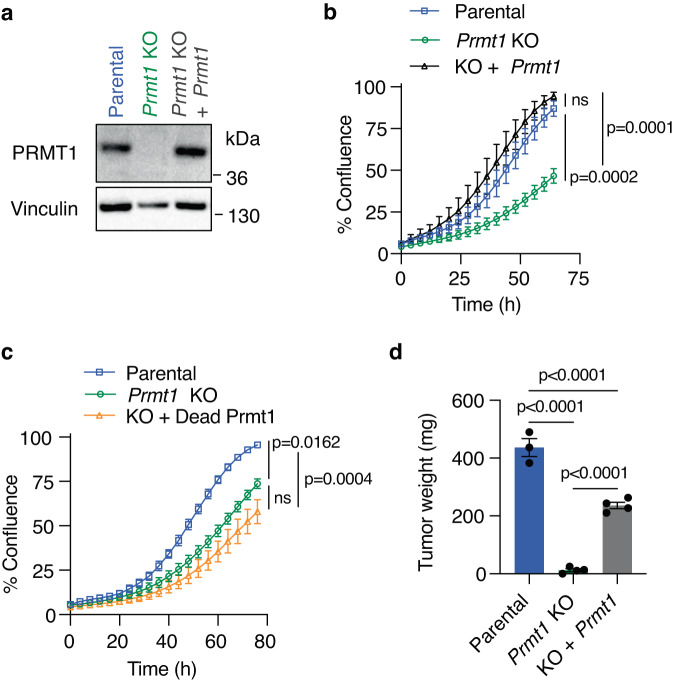
Loss of PRMT1 impedes tumor growth. **a** Representative immunoblot from two independent experiments of Prmt1 levels in FC1245 parental, *Prmt1* KO, or *Prmt1* KO cells rescued with re-expression of exogenous Prmt1. Cell confluence determined using IncuCyte software from phase-contrast images of **b** FC1245 parental, *Prmt1* KO, or *Prmt1* KO cells rescued with re-expression of exogenous wild-type Prmt1 (*n* = 3 independent experiments) and **c** FC1245 parental, *Prmt1* KO, or *Prmt1* KO cells expressing exogenous catalytically dead Prmt1 (*n* = 4 independent experiments). **d** Tumor weights from C57BL/6J mice orthotopically transplanted with FC1245 parental (*n* = 3), *Prmt1* KO (*n* = 4), or *Prmt1* KO cells re-expressing Prmt1 (*n* = 4) and sacrificed 4 weeks after transplant. Data represent means ± SD in (**b**, **c**) and means ± SEM in (**d**). One-way ANOVA with Sidak’s multiple comparisons test was performed in (**b**, **c**) and one-way ANOVA followed by Tukey’s multiple comparison test in (**d**). ns: not significant. Source data are provided as a Source Data file.

As the cellular effects of Prmt1 are in part regulated by hnRNP F, we determined the relative contributions of these two proteins to tumor cell proliferation. CRISPR/Cas9-mediated deletion of *Prmt1* (Fig. 4a) reduced proliferation of FC1245 cells in 2D (Fig. 4b; 38% reduction in confluence at 80 h) as well as in 3D soft agar cultures (Supplementary Fig. 4). Expression of wild-type exogenous *Prmt1* transcript variant 1 at levels similar to those observed in the parental line, but not a catalytically dead version, completely rescued the proliferative defect of the *Prmt1* KO cells (Fig. 4a–c). Notably, the deletion of *Prmt1* largely abrogated tumor growth in orthotopic transplants in syngeneic mice, an effect that was partially rescued by the re-expression of Prmt1 (Fig. 4d).

### Prmt1 regulates global protein translation via Ubap2l asymmetric dimethylation

**Fig. 5:**
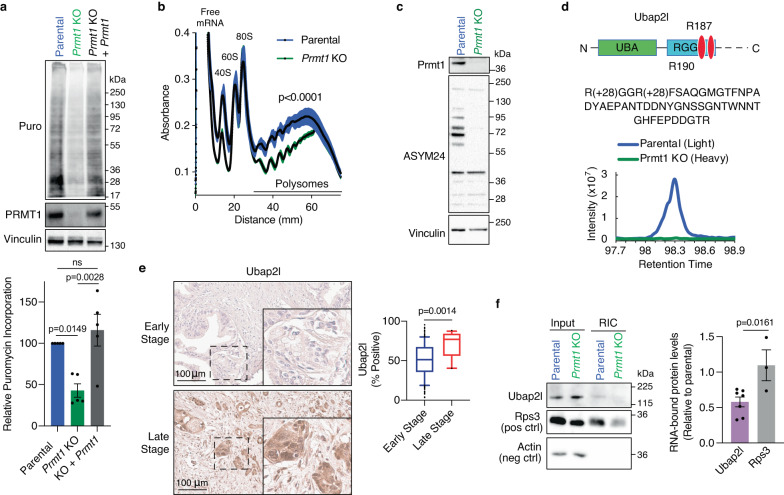
Prmt1 regulates global protein translation via Ubap2l asymmetric dimethylation. **a** Representative immunoblot (top) and densitometric quantification (bottom) of whole cell extracts from puromycin-treated FC1245 parental, *Prmt1* KO, or *Prmt1* KO cells rescued by re-expression of exogenous Prmt1 (*n* = 5 independent experiments). **b** Polysome profiling of FC1245 parental or *Prmt1* KO cells showing the normalized absorbance at 260 nm during fractionation of polysomes (*n* = 3 biological replicates). **c** Representative immunoblot from three independent experiments showing Prmt1 and asymmetrically dimethylated arginine-containing protein (ASYM24) levels in FC1245 parental and *Prmt1* KO cells. **d** (Top) Schematic of UBAP2L protein structure displaying the N-terminal ubiquitin-associated (UBA) domain and the Arg–Gly–Gly (RGG) domain that contains the two Arginines asymmetrically dimethylated by Prmt1 (red ovals). (Bottom) Extracted MS1 chromatographic peak of the listed Ubap2l peptide, showing dimethylation at both R’s in the N-terminal RGGR motif. FC1245 parental peptide was labeled light and the *Prmt1* KO sample was labeled heavy. **e** Representative images (left) and quantification (right) of UBAP2L IHC from a human PDAC tissue microarray containing biological replicates of early (*n* = 149) or late (*n* = 13) stage PDAC. Scale bar: 100 µm. **f** Representative immunoblot (left) and quantification (right) of input and RNA interactome capture (RIC) fractions using antibodies against Ubap2l (*n* = 7 independent experiments) and Rps3 (*n* = 3 independent experiments). Data represent the mean ± SEM in (**a**, **b**) and (**f**). Box plots indicate median (middle line), 25th, 75th percentile (box), 10th and 90th percentile (whiskers), and outliers (single points). One-way ANOVA followed by Dunn’s multiple comparisons test was performed in (**a**), unpaired two-tailed t-test in (**b**) and (**f**), and two-tailed Mann–Whitney test in (**e**). ns: not significant. Source data are provided as a Source Data file.

To explore how Prmt1 regulates tumor growth, we determined the global transcriptional changes induced by the loss of *Prmt1* expression via RNA-seq. Notably, ontology analysis of the downregulated gene set revealed an enrichment of genes involved in protein translation and cell cycle (Supplementary Fig. 5a). Consistent with this, de novo protein translation was reduced by >50% in *Prmt1* KO cells (Fig. 5a), as measured by surface sensing of translation (SUnSET) assays^47^. Importantly, the expression of exogenous Prmt1 in the KO cells rescued this translational defect. Corroborating a role in protein translation, polysome profiling showed that the polysome-associated fraction of mRNA was decreased by nearly 40% in the *Prmt1* KO cells (Fig. 5b).

To provide mechanistic insight into how Prmt1 affects protein translation, we compared the extent of protein methylation in the parental and *Prmt1* KO cells. Western blotting with an antibody that specifically recognizes asymmetric arginine dimethylation, ASYM24^42^, revealed a profound reduction of modified proteins in *Prmt1* KO cells (Fig. 5c). To identify specific targets potentially responsible for the reduction in cell proliferation, quantitative mass spectrometry was performed on modified peptides immunoprecipitated with ASYM24 from trypsinized *Prmt1* KO and parental cell lysates. 9 out of the top 13 identified peptides mapped to the Ubap2l protein (Supplementary Data 2), which we have previously shown to play a key role in the maintenance of global protein synthesis at the post-transcriptional and translational level^48^. Notably, the asymmetric dimethylation of the C-terminal Arg187-Gly-Gly-Arg190 (RGGR) motif of Ubap2l was abrogated in the *Prmt1* KO cells (Fig. 5d; Supplementary Fig. 5b).

A key component of stress granules^49^, *UBAP2L* is amplified in lung adenocarcinoma and breast cancer, where it correlates with poor prognosis^50^ and regulates the expression of cell cycle genes^51^, respectively. Our interrogation of publicly available scRNA-seq data^33,34^ revealed that *UBAP2L* is upregulated in PDAC cells compared to normal ducts and that its expression increases with tumor stage (Supplementary Fig. 5c, d). Further corroborating these results, immunohistochemical evaluation of UBAP2L expression in clinically annotated human PDAC samples revealed that UBAP2L protein levels are also upregulated in late-stage PDAC compared to early-stage (Fig. 5e). Given these findings, we assessed the role of Prmt1-mediated Arginine methylation on UBAP2L function and found that loss of UBAP2L methylation reduced its RNA-binding by >40%, as assessed by RNA interactome capture (RIC) followed by immunoblotting (Fig. 5f). Using a complementary method, we found that a R187A/R190A mutant (referred to as AGGA) that cannot be methylated by Prmt1, also displayed reduced RNA binding (~50%) compared to WT UBAP2L (Supplementary Fig. 5e), corroborating our RIC results.

### Loss of UBAP2L impedes tumor growth by decreasing translation globally

**Fig. 6:**
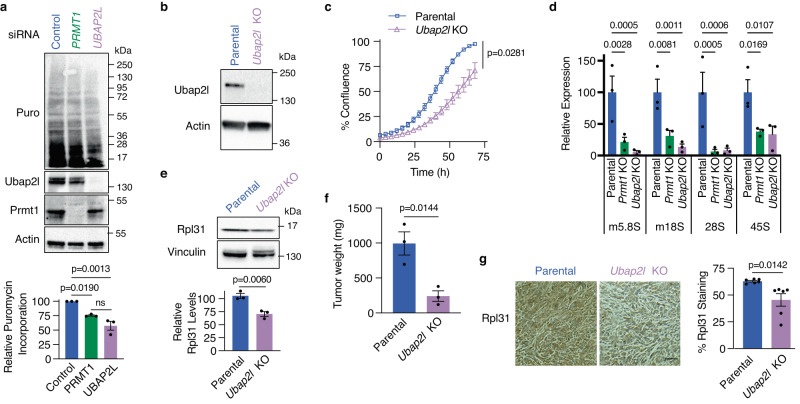
Loss of UBAP2L reduces tumor growth by decreasing global translation. **a** Representative anti-puromycin immunoblot (top) and densitometric analysis (bottom) of extracts from puromycin-treated MIA PaCa-2 cells knocked down using siRNAs against *UBAP2L*, *PRMT1*, or a non-targeting control (*n* = 3 biological replicates). **b** Representative immunoblot from two independent experiments showing Ubap2l levels in FC1245 parental and *Ubap2l* KO cells. **c** Cell confluence determined using IncuCyte software from phase-contrast images of FC1245 parental or *Ubap2l* KO cells (*n* = 3 independent experiments). **d** mRNA expression of the indicated rRNAs, normalized to *Gapdh*, in FC1245 parental, *Prmt1* KO, and *Ubap2l* KO cells (*n* = 3 biological replicates). **e** Representative immunoblot (top) and quantification (bottom) showing Rpl31 protein levels, normalized to Vinculin, in FC1245 parental and *Ubap2l* KO cells (*n* = 3). **f** Tumor weights from mice orthotopically transplanted with FC1245 parental or *Ubap2l* KO cells (*n* = 3). **g** Representative images (left) and quantification (right) of tumor sections of FC1245 parental and *Ubap2l* KO tumors stained with Rpl31 (*n* = 6 from 2 fields per sample; scale bar: 100 μm). Data represent means ± SEM in (**a**, **d**, **e**–**g**), and means ± SD in (**c**). One-way ANOVA followed by Tukey’s multiple comparison test was performed in (**a**), unpaired two-tailed t-test in (**c**) and (**e**–**g**), and two-way ANOVA with Dunnett’s multiple comparison test in (**d**). ns: not significant. Source data are provided as a Source Data file.

To determine the extent to which UBAP2L regulates protein translation downstream of PRMT1 in PDAC, global protein synthesis was compared in MIA PaCa-2 cells after the knockdown of *UBAP2L* or *PRMT1*. SUnSET assays revealed that transient *UBAP2L* knockdown reduced de novo protein translation to a greater extent than *PRMT1* knockdown (~40% and ~25%, respectively; Fig. 6a), consistent with UBAP2L functioning downstream of PRMT1. Importantly, the re-expression of WT UBAP2L, but not the AGGA mutant, in *Ubap2l* KO cells rescued the translational defect, and knockdown of *Prmt1* in AGGA-expressing cells did not further decrease translation (Supplementary Fig. 6a), supporting the role of PRMT1 methylation in UBAP2L-mediated protein translation. Further corroborating these results, *Ubap2l* KO cells re-expressing WT UBAP2L were more sensitive to Prmt1 inhibition compared to those re-expressing the methylation deficient mutant, suggesting that at least part of the Prmt1 effect is mediated through Ubap2l (Supplementary Fig. 6b). Indeed, transcriptomic analysis revealed that 45% of downregulated genes and 39% of upregulated genes upon *Prmt1* knockout are also sensitive to loss of *Ubap2l* (Supplementary Fig. 6c). Together, these data support our hypothesis that Ubap2l acts downstream of Prmt1 to mediate translation.

Functionally, knockout of *Ubap2l* in FC1245 cells (Fig. 6b) decreased cell proliferation (50% reduction in confluence at 72 h; Fig. 6c) and impaired soft agar colony formation (Supplementary Fig. 6d), consistent with protein translation being rate limiting in these cells. Extending from our previous finding of direct ribosomal interactions^48^, we explored whether Ubap2l regulates ribosome biogenesis in PDAC cells. Indeed, the levels of *5.8S*, *18S*, *28S*, and *45S* rRNAs were reduced >60% in *Ubap2l* KO and *Prmt1* KO cells (Fig. 6d). Since our RNA-seq revealed that the large ribosomal subunit polypeptide *Rpl31* and the Eukaryotic Translation Elongation Factor 1 Delta (*Eef1d*) subunit that functions as a guanine nucleotide exchange factor were downregulated in both *Hnrnpf* and *Prmt1* KO cells (Supplementary Fig. 6e), we assessed their protein levels in *Ubap2l* KO cells. Rpl31 and Eef1d levels were decreased in the *Ubap2l* KO cells compared to the parental line (Fig. 6e and Supplementary Fig. 6f). These data indicate that in tumor cells, Ubap2l affects the expression of both rRNAs and ribosomal proteins to potentiate ribosome biogenesis and thereby, global translation.

To explore the relevance of these findings to PDAC, the effect of *Ubap2l* knockout on tumor growth was determined. *Ubap2l* KO led to a >75% reduction in tumor burden when these cells were orthotopically transplanted into the pancreas of syngeneic mice (Fig. 6f). This decrease in tumor burden corresponded with a >25% reduction in RPL31 staining of tumor sections (Fig. 6g), further substantiating a role for UBAP2L in regulating translation in vivo in PDAC.

### Myc coordinates the *Hnrnpf-Prmt1-Ubap2l* network

**Fig. 7:**
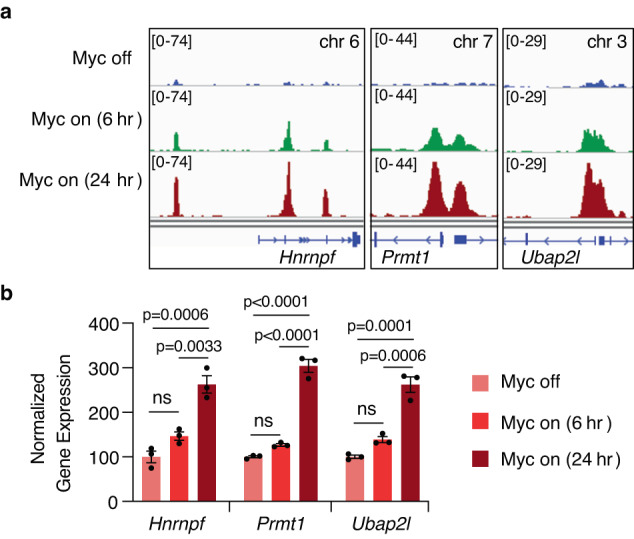
Myc coordinates the *Hnrnpf-Prmt1-Ubap2l* network. **a** Genome browser tracks (from one of two independent experiments) showing Myc binding at the *Hnrnpf*, *Prmt1*, and *Ubap2l* loci upon activation of MycER^T2^ with 4-OHT for the indicated time points. **b** RT-qPCR showing *Hnrnpf, Prmt1*, and *Ubap2l* expression, normalized to *Actb*, in mouse pancreatic epithelial cells in which MycER^T2^ was activated with 4-OHT for the indicated time points. Data represent the mean ± SEM from three biological replicates. One-way ANOVA followed by Tukey’s multiple comparison test was used. ns: not significant. Source data are provided as a Source Data file.

Given the established role of Myc in regulating protein translation^11–13^ and our recent demonstration that Myc is required for progression to adenocarcinoma^52^, we explored a potential role for Myc in the SE-driven network we have uncovered. Interrogation of Myc ChIP-seq data from MIA PaCa-2 cells (GEO accession GSE143804)^53^ revealed MYC binding to the distal *HNRNPF* enhancer as well as the *HNRNPF, PRMT1*, and *UBAP2L* promoters (Supplementary Fig. 7a–c). In agreement with these findings, we show a progressive increase in Myc binding at the *Hnrnpf, Prmt1*, and *Ubap2l* loci in mouse PDAC DF3.4F cells isolated from tumors derived from *pdx1*-*Cre*;*LSL-Kras*^G12D^;*Rosa26LSL-MycER*^T2^ mice^52^ upon induction of quasi-physiologic levels of Myc (Fig. 7a). Moreover, this Myc activation led to progressive increases in *Hnrnpf, Prmt1*, and *Ubap2l* expression levels (Fig. 7b), suggesting that the regulation of translation by Myc is mediated in part, by the coordinated amplification of this *Hnrnpf-Prmt1-Ubap2l* network. These data are further substantiated by analysis of tumor cells from a human PDAC scRNA-seq dataset^54^ that shows a significant correlation between *MYC*, *HNRNPF*, *PRMT1*, and *UBAP2L*, on a cell-by-cell basis (Supplementary Fig. 7d). Further supporting a role for *HNRNPF*, *PRMT1*, and *UBAP2L* in PDAC, these genes are cumulatively amplified in ~28% of PDAC samples^55,56^ (Supplementary Fig. 7e).

### Pharmacologic inhibition of Prmt1 impedes tumor growth

Based on our genetic loss-of-function studies showing reduced tumor burden upon knockout of components of the network we have identified, we postulate that pharmacologic perturbation of this pathway would be therapeutically beneficial in PDAC. Consistent with our *Prmt1* KO cell transcriptomic analysis, pharmacological inhibition of FC1245 KPC cells with selective and potent preclinical small molecule Prmt1 inhibitors PRMT1-2e/TC-E 5003^57^ and AMI-408^58,59^ revealed cell cycle and processes involved in protein translation, such as ribosome biogenesis and rRNA processing, as being impacted (Supplementary Fig. 8a). Indeed, 57% of the differentially expressed genes common to the Prmt1 inhibitors we used can be accounted for by *Prmt1* knockout (Supplementary Fig. 8b). To further validate these inhibitors, we assessed their on-target action through immunoblotting with an antibody that specifically recognizes asymmetric arginine dimethylation. Indeed, Prmt1 inhibition reduced overall asymmetric arginine dimethylation in FC1245 cells (Supplementary Fig. 8c).

**Fig. 8:**
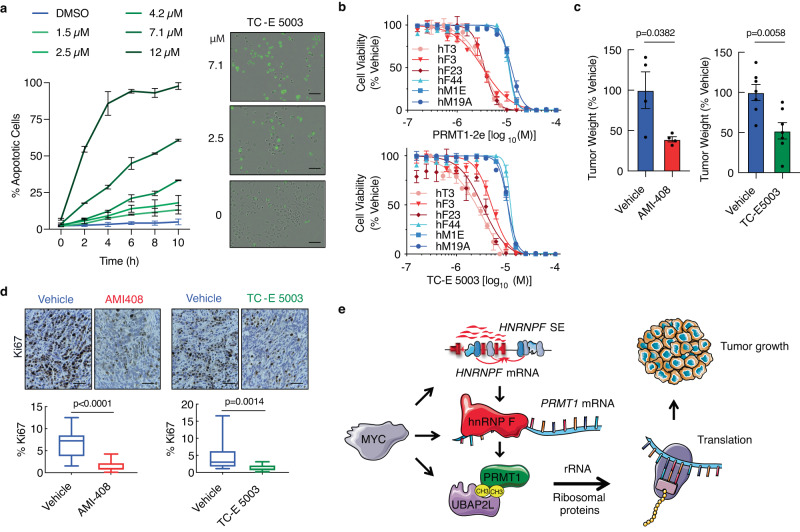
Inhibition of PRMT1 impedes tumor growth. **a** Caspase-3/7 activity assay showing quantification of % apoptotic cells (left) and representative images (right) of MIA-PaCa2 cells treated with the indicated TC-E 5003 doses for 16 h (*n* = 2 independent experiments). The cells labeled in green are undergoing apoptosis. Scale bar: 100 μm. **b** Cell viability assay showing dose-response curves for MYC-high organoids hF3 (*N* = 4), hF23 (*N* = 3), and hT3 (*N* = 3) and MYC-low organoids hM19A (*N* = 4), hM1E (*N* = 4), and hF44 (*N* = 4) treated with the indicated PRMT1 inhibitors for 5 days. **c** Tumor weights, normalized to vehicle, from mice orthotopically transplanted with FC1245 parental cells and treated 10 days post-transplant with AMI-408 or vehicle (*n* = 4) or with TC-E 5003 or vehicle (*n* = 7) for 2 weeks. **d** Representative images (top) and quantification (bottom) of Ki67-stained tumor sections from mice treated with vehicle or AMI-408 (*N* = 16 from 4 fields per sample) or with vehicle or TC-E 5003 (*N* = 14 from 2 fields per sample). Scale bar: 100 µm. **e** Model showing that the *HNRNPF* SE regulates tumor growth by driving the expression of *HNRNPF*. In turn, hnRNP F regulates *Prmt1* mRNA levels. Prmt1 asymmetrically dimethylates Ubap2l, which regulates the expression of rRNA and ribosomal proteins to control protein translation. Increased protein translation leads to increased tumor growth. Myc transcriptionally coordinates this entire program. The figure was partly generated using Servier Medical Art, provided by Servier, licensed under a Creative Commons Attribution 3.0 unported license. Data represent the mean ± SEM in (**a**–**c**). Data in (**a**) represent means and the range. Box plots in (**d**) indicate median (middle line), 25th, 75th percentile (box), minima and maxima (whiskers). One-way ANOVA with Tukey’s multiple comparison test was used to compare IC50s in (**b**). For both PRMT1-2e and TC-E 5003, hM19A vs hF3, hF23 or hT3 *p* < 0.0001, hM1E vs hF3, hF23 or hT3 *p* < 0.0001, hF44 vs hF3, hF23 or hT3 *p* < 0.0001. The remaining comparisons were not significant. An unpaired two-tailed t-test was performed in (**c**) and a two-tailed Mann–Whitney test in (**d**). ns: not significant. Source data are provided as a Source Data file.

To assess whether PRMT1 inhibition is cytotoxic, MIA PaCa-2 cells were treated with TC-E 5003, and caspase-3/7 activity was measured. Indeed, PRMT1 inhibition dose-dependently induced apoptosis in these PDAC cells (Fig. 8a). Encouragingly, PRMT1 inhibition also dose-dependently reduced cell viability of a set of human PDAC organoid lines^60,61^, as measured via CellTiter-Glo assays (Fig. 8b). The different sensitivities of individual organoid lines led to the notion that the efficacy of PRMT1 inhibitors was dependent on MYC expression levels. Indeed, a Pearson correlation revealed an inverse correlation between Myc expression and the IC50 of the PRMT1 inhibitors (Supplementary Fig. 8d). To assess whether Myc activation is sufficient to increase sensitivity to Prmt1 inhibition, FB21.3F mouse pancreatic epithelial cells isolated from tumors derived from *p48*-*Cre*;*LSL-Kras*^G12D^;*Rosa26*^*LSL*^-MycER^T2^ mice were plated in the presence of 4-OHT (Myc on) or EtOH (Myc off). Then, 24 h later, the cells were treated with PRMT1 inhibitors for an additional 24 h. Cells in which Myc was activated, as assessed by upregulation of the Myc target gene *Rasd2* (Supplementary Fig. 8e; TRANFAC Curated Transcription Factor Targets Dataset), were more sensitive to PRMT1 inhibition (Supplementary Fig. 8f), suggesting that Myc-high tumors are sensitized to Prmt1 inhibition.

Given that Myc overexpression occurs in ~40% of PDAC^62^ and that Myc-dependent tumors are particularly sensitive to protein synthesis inhibition^63^, we assessed whether pharmacologic inhibition of Prmt1 would impede tumor growth in vivo. Indeed, the bio-available Prmt1 inhibitors AMI-408^58,59^ (Supplementary Fig. 8c) and TC-E 5003 achieved a 50–60% reduction in tumor burden (Fig. 8c) accompanied by a decrease in Ki67^+^ proliferative cells after 2 weeks of treatment in a syngeneic orthotopic model of PDAC (Fig. 8d). Collectively, these data demonstrate that inhibiting the hnRNP F-Prmt1-Ubap2l pathway has therapeutic potential in PDAC, particularly in Myc-driven tumors.

## Discussion

Directly targeting known oncogenic drivers of PDAC, such as MYC and KRAS, has been historically challenging^4^. Given the highly coordinated programs that drive tumorigenesis, we aimed to uncover epigenetic commonalities and their downstream programs to identify alternative therapeutic approaches. Acknowledging SEs as orchestrators of PDAC pathogenesis, we uncovered an RBP-regulated network that enhances aspects of protein translation, including ribosome biogenesis, to drive tumor growth (Fig. 8e). While tumor cells have been shown to evolve SEs at key oncogenes to enhance signaling pathways^21^, our data demonstrate that SEs also control the expression and function of the protein synthesis machinery required to support these oncogenic processes. While the hnRNP F-Prmt1-Ubap2l network is likely a general mechanism to regulate translation that is operational in normal cells, cancer cells hack into this network, including at the epigenetic level through the SE, to promote tumor growth. Even though we identified the *HNRNPF* SE as a driver of PDAC, the H3K37Ac signal at this locus is elevated in other cancer types compared to normal tissues, suggesting that this pathway is aberrantly activated in other cancers as well. Therapies that disrupt SEs, including bromodomain and extra-terminal (BET) motif inhibitors, have emerged as promising investigational new drugs^64^. However, to date, limited clinical activity and/or severe toxicity has precluded the regulatory approval of such inhibitors^65,66^. By dissecting a particular SE-regulated cascade, we have identified PRMT1 as a downstream druggable target that could be used to intercept SE-driven cancers while potentially avoiding some of the severe toxicities that have emerged from other clinical SE-targeted therapies. This finding of a SE-regulated RBP network that modulates protein synthesis to enable tumor growth elucidates a targetable link between a SE in the nucleus regulating mRNA translation in the cytoplasm.

Deletion of a single SE associated with *HNRNPF* was sufficient to result in a >80% reduction of tumor growth. This alone is important because it reveals that understanding critical SE-regulated networks can uncover essential elements of tumor growth that can be therapeutically targeted. While we cannot exclude the possibility that the *HNRNPF* SE regulates other more distal genes to mediate other cellular functions, *HNRNPF* is the predominant gene responsible for the observed decrease in proliferation in these PDAC cells. Indeed, hnRNP F has been shown to regulate proliferation in other cancer types, including breast^37^, lung^67^, and bladder cancer cells^32^. Moreover, our data provides mechanistic insight, suggesting that besides its well-established role in splicing regulation, hnRNP F also plays a role in stabilizing mRNAs. Indeed, hnRNP F has been shown to regulate *Snai1* mRNA stability in epithelial-to-mesenchymal transition in bladder cancer^35^. While this mRNA is not expressed in our PDAC system, our finding that the stability of nearly 20% of direct hnRNP F targets, including *PRMT1*, are dependent on hnRNP F, suggests a broader role for hnRNP F in PDAC.

Further delineation of this SE-regulated cascade identified PRMT1 as a potential PDAC therapeutic target with available clinical inhibitors. This is in line with previous studies demonstrating the potential utility of perturbing *PRMT1* in PDAC via subcutaneous xenograft models^43,68^. Mechanistically, we demonstrate that Prmt1 affects tumor growth primarily by regulating de novo protein translation though Ubap2l’s control of rRNA and ribosomal proteins. Prmt1 asymmetrically dimethylates Ubap2l within its RNA-binding RGG domain, which we have previously shown is necessary for its interaction with RNA^48^. This is corroborated by proteomic studies that confirm PRMT1-mediated methylation of UBAP2L at the previously identified arginine residues (Fig. 4c, d), and by immunoprecipitation studies that demonstrate a PRMT1-UBAP2L interaction in PDAC cells^69,70^. Of note, *Prmt1* transcript variant 1 fully rescued the in vitro proliferative defect of both *Hnrnpf* and *Prmt1* KO cells. This transcript is predicted to be predominantly cytoplasmic based on its homology to its human ortholog *PRMT1* v2^71^, as is its downstream target Ubap2l^48^. This is in line with other studies that found that this variant, while expressed at a lower level than human *PRMT1* v1, is predominantly responsible for the oncogenic activities of PRMT1 in colon^72^ and breast cancers^73^. Importantly, loss of Prmt1-mediated methylation was sufficient to disrupt Ubap2l translational control by decreasing RNA binding. This is consistent with studies that show that arginine methylation of RBPs affects RNA binding^74^. Encouragingly, the viability of embryonic stem cells derived from *Prmt1* knockout blastocysts^75^ suggests that vulnerabilities arising from the loss of Prmt1 are context dependent. In PDAC, treatment with PRMT1 inhibitors is cytotoxic, consistent with an increased dependency of cancer cells on protein synthesis. While our findings also identify hnRNP F and UBAP2L as potential therapeutic targets in PDAC, there are currently no known tools or clinical compounds that inhibit these RBPs. However, RNA interference-based oligonucleotides or small molecule inhibitors of RBPs have emerged as promising avenues of regulating RBP activity^9^.

Global protein translation is aberrantly activated in numerous cancer types, reflecting the changes in the translation machinery necessary to integrate oncogenic signals and maintain tumor growth^11–13^. In PDAC cells, protein synthesis is elevated both in vitro and in vivo compared to normal tissue, suggesting a dependency on increased translation that may be therapeutically targeted^14,15^. Indeed, overexpression of Myc, which occurs in ~40% of PDAC^62^, leads to an increase in the rate of protein synthesis through its key role in facilitating ribosome biogenesis and protein translation^13,76^, thus sensitizing cells to drugs that interfere with translation, as illustrated in the treatment of multiple myeloma^77^. Our finding that Myc coordinates this *Hnrnpf-Prmt1-Ubap2l* network by regulating all three nodes in this SE cascade exposes an alternative means to target Myc-regulated translation. While reducing Myc levels leads to PDAC regression^52^, efforts to therapeutically modulate Myc have not been successful^4^, and targeting Myc upstream, such as through BET inhibitors, has only a transient impact due to compensatory effects^78^. Here, we show that MYC-high human organoids and mouse PDAC cells in which MycER was activated exhibit an increased sensitivity to PRMT1 inhibition. Additionally, our recent work has revealed UBAP2L as a vulnerability in Myc-amplified triple-negative breast cancer cells^8^. Consequently, targeting oncogene addiction by curtailing de novo protein translation via the Myc-regulated cascade could have therapeutic potential as this vulnerability can be clinically exploited. While MYC-amplified tumors may be more sensitive to translation inhibition, RAS-driven cancers such as PDAC and non-small cell lung cancer also depend on MYC^52,79^, making them promising indications for such a therapeutic approach. Furthermore, targeting the protein synthesis machinery impacts multiple oncogenes simultaneously, which could potentially overcome compensatory mechanisms of resistance. In conclusion, our results provide a druggable molecular mechanism by which tumor cells amplify translation in order to sustain tumorigenesis. This is accomplished at the SE level, allowing cells to mount a coordinated response via the *Hnrnpf-Prmt1-Ubap2l* axis. The concerted control by Myc of these three key interdependent regulators of protein synthesis offers an alternative approach for targeting pancreatic cancer and potentially other cancers.

## Methods

This research complies with all relevant ethical regulations, including the Animal Resources Department at the Salk Institute for Biological Studies, the Salk Institute Institutional Review Board, the Western Institutional Review Board, and the Institutional Animal Care and Use Committee (IACUC).

### Cell lines

The human PDAC cell lines MIA PaCa-2 (CMR-CRL-1420), PANC-1 (CRL-1469), Capan-1 (HTB-79), Capan-2 (HTB-80), Hs766T (HTB-134), PSN1 (CRM-CRL-3211), Panc02.03 (CRL-2553), Panc03.27 (CRL-2549), and Su.86.86 (CRL-1837) were acquired from ATCC and YAPC (ACC 382), HUP-T4 (ACC 223), KCI-MOH1 (ACC 498), PaTU-8902 (ACC 179), and Pa-TU-8988T (ACC 162) were from DSMZ. The KPC mouse PDAC cell line FC1245^80^ and the human PDAC organoid lines hT3^60^, hF3, hF23, hF44, hM1E, and hM19A^61^, were graciously provided by Dr. David Tuveson (CSHL). The primary human PDAC cell line AA0779E^81^ was from Andrew Lowy (UCSD) and the Myc-inducible mouse PDAC cell line DF3.4F^52^ was Gerard Evan (Crick). The FB21.3F Myc-inducible mouse PDAC cell line was isolated from tumor-bearing p48-Cre;LSL-Kras^G12D^; Rosa26^LSL^-MycER^T2^ mice. MUTJ^82^ cells were obtained from the University of Arizona Cancer Center. All cell lines were routinely tested for mycoplasma and tested negative. All cells were maintained frozen until use, so none of the cell lines were further authenticated. KCI-MOH1, is a derivate of the human PDAC cell line HPAC, and as such, we included it in our analysis of human PDAC cell lines without authentication. All cells were cultured according to the supplier’s instructions.

### Animal models

C57BL/6J (Jackson Laboratory Cat #000664, RRID:IMSR_JAX:000664) and NCG (Charles River Laboratories NOD-*Prkdc*^*em26Cd52*^*Il2rg*^*em26Cd22*^/NjuCrl, RRID:IMSR_CRL:572) mice were maintained in a pathogen-free animal facility at the Salk Institute for Biological Studies, following the Institutional Animal Care and Use Committee’s guidelines, on a 12-h light-dark cycle, at an ambient temperature of 23 °C, and 30–70% humidity. Water and food were provided ad libitum.

### Human biopsy

A biopsy (S008) was collected under WIRB 20170433 from a 64-year-old self-reported male. The biopsy was frozen in BamBanker within 30 min of excision and was processed under the Salk Institute for Biological Studies IRB 18-0005. For the biopsy, patients were selected based on their eligibility for the protocol (clinical trial.gov NCT03117920). Biopsy costs were covered by the study, but the patients did not receive additional compensation. The patients gave written informed consent. No sex-based analysis was performed, as only one biopsy is presented in this manuscript.

### Chromatin immunoprecipitation (ChIP)-Seq

For the super-enhancer analysis, ChIP-seq (Supplementary Data 3) was performed on 16 biological replicates of primary and established human PDAC cell lines (AA0779E, MIA PaCa-2, PANC-1, Capan-1, Capan-2, Hs766T, HUP-T4, KCI-MOH1, MUTJ, PSN1, PA-TU-8902, PA-TU-8988T, Panc 02.03, Panc 03.27, Su.86.86, and YAPC) using an H3K27Ac antibody (Abcam Cat# *ab4729*, *RRID:* AB_2118291, 1 µg). Briefly, cells were fixed, nuclei were isolated, lysed, and sheared with a Diagenode Bioruptor to yield DNA fragment sizes of 200–1000 bp, and active chromatin was immunoprecipitated. Libraries were prepped and sequenced on the Illumina HiSeq4000 using barcoded multiplexing and single-end 100 bp length. Reads were aligned using Bowtie2 to GRCh37 and SE peaks were called using HOMER v4.11.1^83^ default settings (-style super, Fold change >4, *p*-value < 0.0001). Data tracks were visualized using IGV v2.3.90.

For the Myc ChIP-seq experiments, mouse PDAC DF3.4F cells isolated from tumors derived from *pdx1*-*Cre*;*LSL-Kras*^G12D^;*Rosa26LSL-MycER*^T2^ mice^52^ were treated with Tamoxifen for 3 weeks. Once the cell line was established, the cells were cultured without 4-Hydroxytamoxifen (4-OHT) for 7 days, after which they were either treated with 100 nmol/L 4-OHT or ethanol control for 6 or 24 h to activate MycER. ChIP-seq was performed as above with the following modifications. One replicate IP was performed using an antibody against c-Myc (Cell Signaling Technology Cat# 9402, RRID:AB_2151827, 1:50) and the other using an antibody against ERα (Santa Cruz Biotechnology Cat# sc-543, RRID:AB_631471, 1 µg). Reads were aligned using Bowtie2 to MGSCv37 (mm9) and differential peaks were called using HOMER’s^83^ default settings (Fold change >4, *p*-value < 0.0001) using ‘-style factor’.

### Flow cytometry

The tumor was dissociated, and flow cytometry was used to separate and collect nuclei from aneuploid epithelial tumor cells^38^. Tissue was minced with scalpels in a solution of 10 µg/ml 4,6-diamidino-2-phenylindole (DAPI) and 0.1% Nonidet P-40 detergent in a Tris-buffered saline. The supernatant was triturated with a 26-gauge needle, filtered through 40 µm steel mesh, and analyzed using an InFlux cytometer (Cytopeia Beckton-Dickenson, Seattle WA), with ultraviolet excitation and DAPI emission collected at >450 nm. DNA content was analyzed MultiCycle (Phoenix Flow Systems, San Diego, CA). 50k aneuploid nuclei were collected in nuclei storage buffer (50 mM Tris·Cl, pH 8.0, 5 mM MgCl_2_, 40% Glycerol, 0.1 mM EDTA) and snap-frozen for ATAC-seq.

### ATAC-seq

ATAC-seq libraries were prepared according to published methods^84^. Libraries were sequenced on an Illumina HiSeq2500 using barcoded multiplexing and a paired-end 42 bp length. Reads were aligned using Bowtie2 to GRCh37 and SE peaks were called using HOMER’s^83^ default settings (-style super, Fold change >4, *p*-value < 0.0001). Data tracks were visualized using IGV v2.3.90.

### Copy-number analysis

DNA was DNAse I digested, labeled using a BioPrime Labeling Kit (Invitrogen) using Cy-5 dUTP for the sample and Cy-3 dUTP for the reference genome, hybridized to 400k comparative genomic hybridization (CGH) arrays (Agilent Technologies), scanned using an Agilent 2565C DNA scanner, and the images were analyzed with Agilent Feature Extraction v11.0 using default settings.

### Generation of CRISPR KO, rescue, and SE deletion cell lines

FC1245 cells were edited by CRISPR/Cas9 to generate KO clones. Plasmid vectors expressing hSpCas9 (PX458 or PX459, Addgene) and chimeric guide RNAs (sgRNAs) were used for cloning of CRISPR/Cas9 targeting constructs and inserts were verified by Sanger sequencing. The sgRNAs (Supplementary Data 4) were designed using publicly available software tools E-CRISPR v5.3 and CHOPCHOP v3^85,86^ and two adjacent sgRNAs were transiently expressed simultaneously to increase the odds of a deletion. The rescue cell lines were generated by stably re-expressing, in the respective FC1245 KO cell lines, a human *HNRNPF* (GeneCopoeia EX-F0678-Lv206) or mouse *Prmt1* (GeneCopoeia EX-Mm03133-Lv206) plasmid containing an mCherry-IRES-Puro cassette driven by an independent promoter. The expression levels in the rescue cell lines were determined empirically. Puromycin was utilized to select a stably-expression population of rescued cells. The *HNRNPF* SE was deleted in MIA PaCa-2 cells by introducing double-stranded breaks at either end of the distal SE (using a similar method as above) and introducing a Hygromycin cassette flanked by Lox-Stop-Lox sites (LSL-Hygro-LSL) in the SE’s place via homology arms 5’ and 3’ of the enhancer. Homology arms were PCR’ed (Supplementary Data 4) from a human BAC (BACPAC Genomics Cat# RP11-260O9). The plasmid containing the LSL-Hygro-LSL cassette was assembled using gBlocks Gene Fragments (IDT) that were cloned into the pFNF backbone (22687, Addgene) by ligation and IN-Fusion cloning (Clontech). The Neomycin resistance cassettes were replaced with a Hygromycin cassette to render the *HNRNPF* SE Homology Arms LSL-Hygro-LSL plasmid. The correct plasmid sequence was confirmed by Sanger sequencing. The LSL-Hygro-LSL cassette was subsequently removed from the MIA PaCa-2 *HNRNPF* SE deleted cells using an Adeno-Cre virus (Ad5CMVCre-eGFP, WC-U of Iowa-1174). *HNRNPF* was knocked out in MIA PaCa-2 cells and in the *HNRNPF* SE deleted MIA PaCa-2 cells using sc-401892 and sc-401892-HDR according to Santa Cruz protocols. The human UBAP2L pENTR clone^48^ was Gateway cloned into pDEST-HA^87^. Prmt1 and UBAP2L mutant clones (Supplementary Data 4) were generated via QuikChange mutagenesis. All generated cell lines are available upon request.

### Quantitative RT-PCR

Total RNA was purified following Trizol extraction according to the manufacturer’s instructions. cDNA synthesis was carried out using 1 μg RNA and iScript Reverse Transcription Supermix (Bio-Rad Cat #1708841), and RT-qPCR was performed using Advanced Universal SyBr Green Supermix (Bio-Rad Cat #725271) on the CFX384 detection system (Bio-Rad). RT-qPCR was carried out in technical triplicates and biological replicate samples were analyzed using Bio-Rad CFX Maestro software v2.3. Biological replicates were averaged to generate mean fold changes and values expressed as fold differences to control samples calculated using the ΔΔC_t_ method. Primer sequences can be found in Supplementary Data 4.

### Immunoblotting

Cells were lysed in RIPA buffer (Sigma) supplemented with Halt protease inhibitor cocktail (Thermo Fisher). Whole-cell lysates were analyzed by SDS-PAGE and immunoblotting. Briefly, lysates were fractionated on 4–12% Bis-Tris gels (Invitrogen), which were then transferred to PVDF membranes and blocked in 5% milk for an hour. The blots were incubated with primary antibodies overnight at 4 °C and secondary HRP-conjugated antibodies for 2 h at room temperature. The following antibodies were utilized: human hnRNP F (Santa Cruz Biotechnology Cat# sc-32309, RRID:AB_627732, 1:1000), mouse/human hnRNP F (Abcam Cat# ab50982, RRID:AB_880477, 1:1000), PRMT1 (R and D Systems Cat# AF6016, RRID:AB_1964684, 1:1000), Vinculin (R and D Systems Cat# MAB6896, RRID:AB_10992930, 1:1000), human UBAP2L (Bethyl Cat# A300-533A, RRID:AB_477953, 1:10,000), mouse/human UBAP2L (Bethyl Cat# A300-534A, RRID:AB_2272582, 1:5000), Anti-dimethyl-Arginine Antibody, asymmetric (Millipore, at# 07-414, RRID:AB_310596, 1:1000), *β*-Actin D6A8 (Cell Signaling Technology Cat# 8457, RRID:AB_10950489 1:1000), RPL31 (ABclonal Cat# A17527, RRID:AB_2772081, 1:1000), EEF1D (ABclonal Cat# A2509, RRID:AB_2764400, 1:1000), anti-rabbit IgG-HRP (Santa Cruz Biotechnology Cat# sc-2004, RRID:AB_631746, 1:10,000), anti-mouse IgG-HRP (Santa Cruz Biotechnology Cat# sc-2005, RRID:AB_631736, 1:10,000), anti-goat IgG-HRP (Millipore Cat# 401515-2ML, RRID:AB_10682600, 1:10,000). Signal was detected by SuperSignal West Pico Chemiluminescent Substrate (Thermo Fisher) using a ChemiDoc Imaging system (Bio-Rad). Uncropped and unprocessed scans are provided in the Source Data file or in the Supplementary Information.

### Cell proliferation assay

For this, 3000 cells/well were plated in three or more replicates in 96-well plates and scanned every 4 h on the IncuCyte S3 v2019A system (Essen BioScience). Percent cell confluence was determined using phase-contrast images.

### Anchorage-independent growth in soft agar

Cells were seeded in 0.4% Bacto Agar at 3000–3500 cells/well in 12-well plates, on top of a 0.6% agar layer. Media was changed every 4 days. Cells were fixed in ice-cold methanol for 20 min after 18 (MIA PaCa-2 cells) or 25 (FC1245 cells) days and stained with crystal violet (0.005% in H_2_O, 20% (v/v) methanol) for 2.5 h at room temperature. The number of colonies with a diameter ≥100 μm was quantified from the entire well using ImageJ 2.0 (National Institutes of Health).

### Orthotopic transplants

For the syngeneic PDAC model, 100 FC1245 cells, isolated and expanded from a female KPC mouse, were orthotopically injected into 8- to 10-week-old age-matched male C57BL/6 mice (Jackson Laboratory Cat #000664, RRID:IMSR_JAX:000664) in 50% complete media, 50% Matrigel. Only male mice were used for these studies to avoid hormonal fluctuations that might impact tumor growth in female mice. However, our findings are applicable to both males and females as the super-enhancers were identified in a mixed population of male and female PDAC cell lines, our organoid experiments included both male and female-derived lines, and our in vivo experiments used both male (MIA PaCa-2) and female (FC1245) cell lines to derive tumors. For the *Hnrnpf* KO experiments, 5 mice were successfully transplanted with the KO cells and 4 mice with the parental cells. For the *Prmt1* KO experiments, 4 mice were successfully transplanted with the KO cells, 4 mice with the rescue cells, and 3 mice with the parental cells. For the *Ubap2l* KO experiments, 3 mice were successfully transplanted with the KO cells and 3 with the parental cells. As our IACUC protocol does not limit tumor size, tumors were monitored for growth by palpation and mice were monitored for changes in body weight and signs of morbidity associated with tumor growth in order to humanely euthanize them via CO_2_ if they reach end-stage criteria. Mice were sacrificed to assess tumor burden 4 weeks after transplantation via CO_2_ asphyxiation. Pancreatic tumors were collected, weighed, and fixed in formalin. For the PRMT1 inhibitor experiments, 10 days after transplantation of FC1245 cells, mice were randomized into two treatment groups: PRMT1 inhibitors TC-E 5003 (8 mg/kg IP, Cayman Chemical Cat# 17718; CAS: 17328-16-4; 7 mice per group) or AMI-408 (1 mg/kg IP, Inotation Research Laboratories; 4 mice per group) and the appropriate vehicle controls. AMI-408 and TC-E 5003 were resuspended in DMSO and freshly diluted in 1:1 PEG300/D5W and administered daily. For the orthotopic xenografts, 500,000 MIA PaCa-2 wild-type or *HNRNPF* SE deleted cells were orthotopically transplanted into male 9-week-old (*n* = 5 each) triple immunodeficient NCG MICE (Charles River Laboratories NOD-*Prkdc*^*em26Cd52*^*Il2rg*^*em26Cd22*^/NjuCrl, RRID:IMSR_CRL:572) and sacrificed after 4 weeks. All procedures involving animals were performed in accordance with protocols approved by the IACUC and Animal Resources Department of the Salk Institute for Biological Studies (protocol # 11-00032).

### Immunohistochemistry

Mouse tumor tissue was fixed in 10% Neutral buffered formalin overnight at 4 °C, rinsed in 70% EtOH, and paraffin-embedded. The human PDAC tissue microarray utilized for hnRNP F IHC staining was from Tissue Array (PA961f) and the one utilized for UBAP2L IHC was from Biomax (PA1921a). Briefly, FFPE tissue sections were deparaffinized and antigen retrieval was performed using a sodium citrate buffer. Staining was performed with antibodies against Ki67 (GeneTex Cat# GTX16667, RRID:AB_422351; 1:50), RPL31 (Abclonal Cat# A17527, RRID:AB_2772081; 1:200), hnRNP F (Mybiosource Cat# MBS178697, 1:1000), or UBAP2L (Sigma-Aldrich Cat# HPA035068, RRID:AB_10696366, 1:500). Slides were stained with diaminobenzidine (DAB) chromogen and counterstained with Hematoxylin. Images were acquired on the Revolve microscope (Echo Laboratories) and the percentage of Ki67-positive or RPL31-positive cells was analyzed using ImageJ 2.0. The tissue microarray images were analyzed using Aperio ImageScope (v12.4.6.5003).

### RNA-sequencing and analysis

Total RNA was isolated using Trizol (Invitrogen) and the RNeasy mini kit with on-column DNase digestion (Qiagen Cat #74106). Sequencing libraries were prepared from 100–500 ng total RNA using the TruSeq RNA Sample Preparation Kit v2 (Illumina Cat# RS-122-2001, RS-122-2002) according to the manufacturer’s protocol. RNA-seq libraries were prepared from two to three biological replicates and sequenced on an Illumina HiSeq4000 using barcoded multiplexing and a single-end 100 bp read length. For the *HNRNPF* knockdown experiment in AA0779E cells, triplicate samples were sequenced on an Illumina HiSeq2500 using a paired-end 75 bp read length. Read alignment and junction mapping to genome build GRCm38 or GRCh38v32 was accomplished using STAR v2.7.1a^88^ or TopHat2 v2.0.4 followed by differential gene expression analysis using Cuffdiff v2.2.1^89^ and the Ensembl genome annotation or DESeq2 v1.30.1^90^. Gene ontology analyses were performed using Metascape (metascape.org) or DAVID v6.8 (https://david.ncifcrf.gov/). For gene expression comparisons between normal pancreas and PDAC, data were obtained from TCGA (https://tcga-data.nci.nih.gov) and GTEx (gtexportal.org) data portals. Non-PDAC tumor and normal samples^91^ were excluded from the TCGA-TAAD cohort. Myc expression for the organoid cell lines was obtained from a previously published dataset^61^. For splicing detection, the vast-tools (v 2.0.2) were used.

To compare the expression of *HNRNPF*, *PRMT1* and *UBAP2L* between untreated PDAC cells and normal ducts, the scRNA-seq data sets^34^ (PRJCA001063) were established using the zendo link: https://zenodo.org/record/6024273#.Yg2eTJZUtaY^33^. After the establishment of the Seurat object, ductal tumor cells were extracted. To compare the expression of *HNRNPF*, *PRMT1* and *UBAP2L* across PDAC stages, we utilized single nuclei RNA-seq data sets from GSE202051^92^. The metadata was matched from the original paper. In order to correlate the expression of *MYC*, *HNRNPF*, *PRMT1*, and *UBAP2L* within the ductal clusters on a cell-by-cell basis, the primary tumor scRNA-seq samples from GSE154778^54^ were utilized. The Seurat object was established using expression data and normalized for integration. One to fifty PCA dimensions were used. Briefly, for all these data sets, the R package Harmony was utilized to integrate samples with the default settings, and the Alra imputation algorithm was used to restore the expression of genes^93^. To extract the count or expression level of each cell, FetchData and AverageExpression were utilized. The violin plots and heatmap were generated in Prism v9.0.

### eCLIP

eCLIP was performed on 1 × 10^7^ cells using the eCLIP Library Prep Kit (Eclipse Bioinnovations Cat# ECEK-0001) according to manufacturing instructions. Membrane regions spanning from the hnRNP F band (~46 kDa) to the first non-specific band noted in the IgG control (~82 kDa) were utilized. Libraries were sequenced on an Illumina HiSeq2500 using dual barcoded multiplexing and a paired-end 75 bp length. Peaks were called using the standard eCLIP processing protocol 0.2, which is available at: https://github.com/YeoLab/eclip.

### siRNA knockdown

Knockdowns were performed using DharmaFECT 1 (Dharmacon) transfection reagent according to the manufacturer’s instructions. Dharmacon ON-TARGETplus human siRNA SMARTpool against *HNRNPF* (L-013449-01-0005), *PRMT1* (L-010102-00-0005), *UBAP2L* (L-021220-01-0005) or Non-Targeting pool (D-001810-10-05) were used. For mouse cells, Accell Mouse *Prmt1* siRNA (A-049497-14-0005) from Horizon Discovery was used. Cells were harvested 3–4 days post knockdown.

### Luciferase reporter assay

The 3’UTR of human *PRMT1* was subcloned at the C-terminus of a firefly luciferase cassette into pMirTarget (SC202712, Origene), which also contained an RFP cassette driven by an independent promoter. This construct was transiently transfected into FC1245 parental, *Hnrnpf* KO, or *Hnrnpf* KO + *Hnrnpf* rescue cells using Lipofectamine 3000. Then, 48 h after transfection, RNA was isolated, and RT-qPCR was performed to calculate the ratio of luciferase to RFP (primer sequences in Supplementary Data 4).

### Organoids

Human PDAC organoids were grown according to established protocols^60,61^, dissociated with TrypLE into single cells and small clumps, and plated in 384-well plates at 1000 viable cells/well in 20 μL slurry of 10% Matrigel and human complete organoid media. PRMT1 inhibitors PRMT1-2e (Xcessbio Cat# M60181-5s; CAS: 17328-16-4) and TC-E 5003 (Cayman Chemical Cat# 17718; CAS: 17328-16-4) were added up to 48 h post plating using an HP D300e Digital Dispenser and organoid viability was measured 5 days later using the CellTiter-Glo luminescence-based viability assay (Promega Cat #G7572) according to the manufacturer’s instructions.

### Caspase-3/7 assay

For this, 3000 MIA-PaCa2 cells were plated per well in 96-well plates. The following day, cells were treated with the indicated TC-E 5003 concentrations and the IncuCyte Caspase-3/7 Green Apoptosis Assay Reagent (Essen BioScience Cat #4440) was added according to the manufacturer’s instructions, using an HP D300e Digital Dispenser. Images were acquired on the IncuCyte S3 v2019A system (Essen BioScience) every 2 h.

### SUnSET assay

De novo global protein synthesis was measured via the SUnSET method^47^. Cells were treated with puromycin (10 µg/ml) for 10 min and then lysed in RIPA buffer containing Halt protease inhibitor cocktail (Thermo Fisher) and cycloheximide (100 µg/ml). Equal amounts of protein were analyzed by immunoblotting, as described above, using an anti-puromycin antibody (Millipore Cat# MABE343, RRID:AB_2566826, 1:10,000) and anti-Mouse IgG2a-HRP Secondary Antibody (Thermo Fisher Scientific Cat# M32207, RRID:AB_2536640, 1:10,000).

### Polysome profiling

Polysome profiling was performed in triplicate from 3 ×15 cm plates, at 60–80% confluence. Cells were treated with cycloheximide (CHX) at 100 μg ml^−1^ for 5 min at 37 °C. The culture medium was removed, and cells were washed twice with cold PBS containing 100 μg ml^−1^ CHX and snap-frozen. Cells were lysed by trituration through a 27-gauge needle in 400 μl polysome lysis buffer (20 mM Tris-HCl (pH 7.4), 150 mM NaCl, 5 mM MgCl_2_) with 1× protease inhibitor cocktail (EMD Millipore), 100 μg ml^−1^CHX, 1 mM DTT, 25 U ml^−1^ DNase (TURBO DNase; Thermo Fisher) and 20 U ml^−1^ RNase inhibitor (RNaseOUT; Thermo Fisher) and incubation on ice for 30 min. Lysates were clarified by centrifugation at 17,500×*g* at 4 °C for 5 min.

### Mass spectroscopy

The PTMScan Asymmetric Di-Methyl Arginine Motif [adme-R] Kit (Cell Signaling Cat# 13474) was used to immunoprecipitate arginine-asymmetrically dimethylated peptides from 11 ×15 cm tissue culture plates at 85% confluence, according to the manufacturer’s instruction. The enriched samples were labeled with dimethyl 30.0439 as the light channel (WT) and 36.0757 as the heavy channel (KO). The labeled samples were pooled and analyzed by LCMS on a Fusion Lumos mass spectrometer (Thermo). The digest was injected directly onto a 30 cm, 75 μm ID column packed with BEH 1.7 μm C18 resin (Waters). Samples were separated at a flow rate of 400 nl/min on a nLC 1200 (Thermo). Solutions A and B were 0.1% formic acid in water and 0.1% formic acid in 90% acetonitrile, respectively. A gradient of 1–30% B over 100 min, an increase to 50% B over 20 min, an increase to 90% B over 10 min and held at 100% B for a final 10 min was used for 140 min total run time. The column was re-equilibrated with 20 μl of A prior to the injection of the sample. Peptides were eluted directly from the tip of the column and nanosprayed directly into the mass spectrometer by application of 2.5 kV at the back of the column. The Lumos was operated in a data-dependent mode. Full MS scans were collected in the Orbitrap at 120 K resolution with a mass range of 400 to 1500 m/z and an AGC target of 4e^5^. The cycle time was set to 3 s, and within these 3 s, the most abundant ions per scan were selected for HCD MS/MS with an AGC target of 4e^5^ and 15 K resolution. Maximum fill times were set to 50 ms and 100 ms for MS and MS/MS scans, respectively. Quadrupole isolation at 1.6 m/z was used, monoisotopic precursor selection was enabled, and dynamic exclusion was used with an exclusion duration of 5 s. Protein and peptide identification were done with Integrated Proteomics Pipeline—IP2 (Integrated Proteomics Applications). Tandem mass spectra were extracted from raw files using RawConverter^94^ and searched with ProLuCID^95^ against the Uniprot mouse database. The search space included all fully tryptic and half-tryptic peptide candidates, carbamidomethylation on cysteine was considered a static modification, methylation and dimethylation were considered variable modifications on arginine. Heavy and light dimethyl labeling were considered modifications on the N-terminus and lysine. Data was searched with 50 ppm precursor ion tolerance and 600 ppm fragment ion tolerance. Identified proteins were filtered to 10 ppm precursor ion tolerance using DTASelect^96^ and utilizing a target-decoy database search strategy to control the false discovery rate to 1% at the peptide level^97^. The modified peptides of Ubap2l were manually validated and dimethyl quantitation was done with Skyline^98^.

### RNA interactome capture (RIC)

UV crosslinked cell pellets were resuspended in ice-cold lysis buffer (20 mM Tris-HCl, pH 7.5, 500 mM LiCl, 1 mM EDTA, 5 mM DTT, 0.5% wt/vol LiDS, 5 mM DTT and complete protease inhibitor cocktail) and incubated on ice for 5 min. Cells were sonicated with Bioruptor Pico (Diagenode) for 30 s on and 30 s off for a total of 5 min. Insolubles were removed by centrifugation at 15,000×*g* for 5 min. Oligo dt beads (NEB) were added and incubated in lysate for 1 h at 37 °C with gentle rotation. Beads were collected with a magnet, and the supernatant was transferred to a new tube for a second round of capture. Beads were then subject to successive rounds of washes using wash buffers 1–3 (buffer 1: 20 mM Tris-HCl, 500 mM LiCl, 1 mM EDTA, 5 mM DTT, and 0.5% LiDs; buffer 2: 20 mM Tris-HCl, 500 mM LiCl, 1 mM EDTA, 5 mM DTT, and 0.1% LiDs; buffer 3: 20 mM Tris-HCl, 200 mM LiCl, 1 mM EDTA, 5 mM DTT, and 0.02% LiDs) with 5 min of gentle rotation. RNA-protein interactions were eluted off the beads using RNAse-free water and combined with 10× RNase buffer, 1 M DTT, and 1% NP40 (final concentrations: 1× RNase buffer, 5 mM DTT, 0.01% NP40) and ∼200 U RNase T1 and RNase A (Sigma-Aldrich). RNA was digested for 60 min at 37 °C. Eluted proteins were resuspended in 2x NuPage LDS running buffer + DTT and run on SDS-page and transferred to nitrocellulose for immunoblotting with antibodies against the protein of interest (Ubap2l), a positive control (Rps3), or a negative control (Actin).

### RNA-binding assay

For isolation and visualization of HA-UBAP2L-bound RNA, we followed the procedure described in Blue et al.^99^. Briefly, cells (~2 × 10^7^) transiently transfected with either WT or R187A/R190A HA-UBAP2L or empty vector, were irradiated with UV light (254 nm, 400 mJ/cm^2^) to induce RNA-protein cross-links, collected, and lysed in eCLIP lysis buffer. Lysates were incubated with 50 µl anti-HA magnetic beads (Pierce, Cat #88837) for 16 h at 4 °C with rotation. Beads were washed, and bound RNA was dephosphorylated on-bead using alkaline phosphatase and polynucleotide kinase. Bound RNA was then 3’-ligated on-bead to biotinylated cytidine (bis)phosphate (pCp-biotin; Jena Bioscience cat# NU-1706-BIO) for 16 h at 16 °C with agitation. After washing the beads, protein-RNA complexes were released by heating in 1× LDS sample buffer (Thermo Fisher) containing 0.1 M DTT for 10 min at 70 °C with agitation, run on a 4–12% NuPAGE bis-tris gel (Thermo Fisher), and transferred to nitrocellulose membrane (Amersham Protran) in 1× NuPAGE transfer buffer (Thermo Fisher) containing 10% methanol. HA-tagged UBAP2L was detected with mouse anti-HA antibody (Abcam Cat# ab49969, RRID:AB_880330, 1 µg/ml) followed by IRDye 800CW goat anti-mouse IgG secondary antibody (LI-COR Biosciences Cat# 926-32210, RRID:AB_621842, 0.1 µg/ml). Biotinylated RNA was detected using IRDye 670RD Streptavidin (LI-COR cat#926-68079, 1:10,000 dilution) and visualized and quantified on an Odyssey Imaging System (LI-COR).

### Quantification and statistical analyses

All statistical details of experiments are included in the figure legends or specific “Methods” sections. Western blot band intensities were quantified with Image Lab 5.2.1, ImageJ 2.0, or Fiji v2.0.0-rc-43/1.52n and normalized to internal controls. All statistical tests were performed using Prism software version 9.0 for mac OS X (GraphPad Software) unless otherwise noted. The *p*-value in the correlation between H3K27Ac signal at the *HNRNPF* SE and *HNRNPF* expression in Fig. 1d was obtained by calculating the z-score from the R^2^ values obtained from the correlation of 1000 randomly selected genes with an average RPKM >10.

### Reporting summary

Further information on research design is available in the Nature Portfolio Reporting Summary linked to this article.

### Supplementary information

Supplementary Information

Description of Additional Supplementary Files

Supplementary Data 1

Supplementary Data 2

Supplementary Data 3

Supplementary Data 4

Reporting Summary

### Source data

Source Data

## Data Availability

The publicly available scRNA-seq data sets used in this study are available in the National Genomics Data Center under accession code PRJCA001063^34^ and the Gene Expression Omnibus (GEO) database under accession codes GSE202051^92^ and GSE154778^54^. The publicly available Myc ChIP-seq data used in this study are available under accession codes GSE143804^53^. Normal pancreas H3K27Ac ChIP-seq data were retrieved from GSM1013129 and GSM906397. Normal cell H3K27Ac ChIP-seq data from 293T, NHEK, myoblasts, monocytes, bronchial epithelial cells, skeletal myotubes, keratinocytes, and macrophages were downloaded from GSM2171416^100^, GSM1666386, GSM4143867, GSM3462802, GSM3892733, GSM3611923, GSM1645725, and GSM1327358, respectively. H3K27Ac ChIP-seq data from cancer cell lines K562, MCF7, He-La-S3, HepG2, Dnd41, A549, and HCT-116 H3K27Ac ChIP-seq were retrieved from the ENCODE database^101^ (GSM733656, GSM945854, GSM733684, GSM733743, GSM1003462, GSM1003578, and GSM945853, respectively). PANC-1 BRD4 ChIP-seq data were retrieved from PRJEB27863^102^. The RNA-seq, ChIP-seq, ATAC-seq and eCLIP data reported in this paper have been deposited in the National Center for Biotechnology Information (NCBI) Sequence Read Archive (SRA) database, under accession codes PRJNA678286 and GSE234078. The mass spectrometry proteomics data are deposited in the ProteomeXchange Consortium via the PRIDE partner repository with the dataset identifier PXD030423. The remaining data are available within the article, Supplementary Information, Supplementary Data, or Source Data file. Source data are provided with this paper.

## Code availability

The current processing pipeline for eCLIP can be found at https://github.com/yeolab/eclip.
