# Supplementary material for: A super-enhancer-regulated RNA-binding protein cascade drives pancreatic cancer
Source: Nat Commun. 2023 Sep 6;14:5195. doi: 10.1038/s41467-023-40798-6 (PMC10482938; doi:10.1038/s41467-023-40798-6)
Supplement: Supplementary file 1 — Supplementary Information [file 41467_2023_40798_MOESM1_ESM.pdf]

SUPPLEMENTARY INFORMATION

A super-enhancer regulated RNA-binding protein cascade drives pancreatic cancer

Corina E. Antal, Tae Gyu Oh, Stefan Aigner, En-Ching Luo, Brian A. Yee, Tania Campos, Hervé Tiriac, Katherine L. Rothamel, Zhang Cheng, Henry Jiao, Allen Wang, Nasun Hah, Elizabeth Lenkiewicz, Jan C. Lumibao, Morgan L. Truitt, Gabriela Estepa, Ester Banayo, Senada Bashi, Edgar Esparza, Ruben M. Munoz, Jolene K. Diedrich, Nicole M. Sodir, Jasmine R. Mueller, Cory R. Fraser, Erkut Borazanci, David Propper, Daniel Von Hoff, Christopher Liddle, Ruth T. Yu, Annette R. Atkins, Haiyong Han, Andrew M. Lowy, Michael T. Barrett, Dannielle D. Engle, Gerard I. Evan, Gene W. Yeo, Michael Downes[*], Ronald M. Evans[*]

SUPPLEMENTARY FIGURES 1-8

Supplementary Figure 1. ***HNRNPF*** **expression and super-enhancer in PDAC**

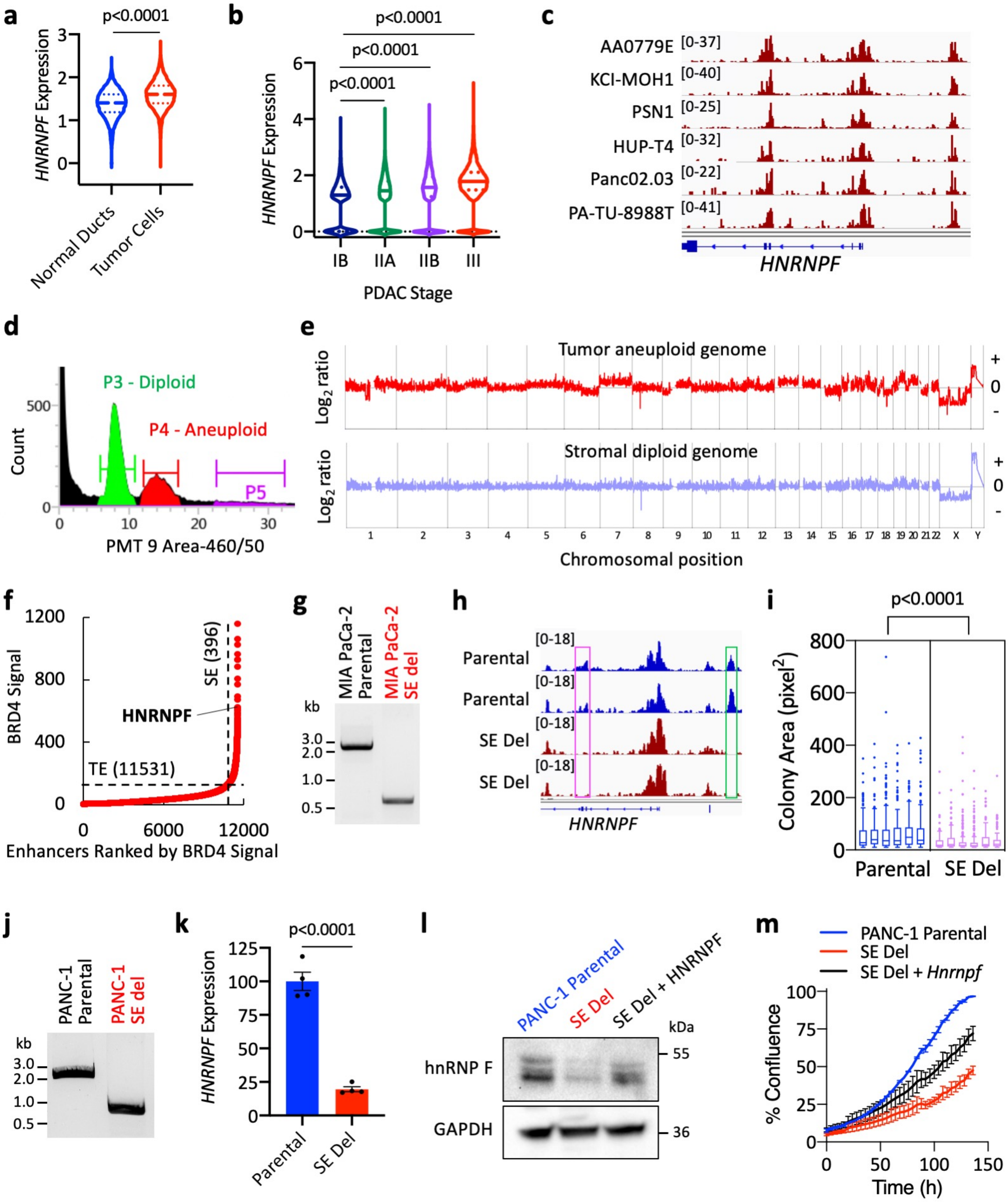

Supplementary Figure 1. *HNRNPF* expression and super-enhancer in PDAC

Violin plots showing scRNA-seq *HNRNPF* expression from (**a**) normal human pancreatic ducts (n=7678 cells) and malignant PDAC cells (n=13917 cells), and (**b**) malignant PDAC cells from stage IB (n=7768 cells), IIA (n=10792 cells), IIB (n=27947 cells), and III (n=17134 cells). **c** Browser tracks showing open chromatin at the *HNRNPF* locus in the indicated PDAC lines. **d** Graph showing DNA content with diploid and aneuploid peaks that were sorted from PDAC tumor biopsy S008. **e** Copy number variation analysis of the stromal diploid and tumor aneuploid populations from tumor biopsy S008. The x- and y- axes in the comparative genomic hybridization plots represent chromosome and $\log_2$ ratios for each genome. **f** Super-enhancers (SE) and typical enhancers (TE) plotted based on their input-normalized BRD4 ChIP-seq signal. The number of enhancers is indicated in parentheses. **g** PCR spanning the distal *HNRNPF* enhancer. **h** Genome browser tracks showing open chromatin at the *HNRNPF* locus in MIA PaCa-2 and SE deleted cells. The green bar indicates the distal SE element that was deleted and the pink indicates the proximal SE element. **i** Quantification of soft agar colony area from MIA PaCa-2 parental and *HNRNPF* SE deleted cells (n=6). **j** PCR spanning the distal *HNRNPF* enhancer. **k** RT-qPCR showing *HNRNPF* expression, normalized to *GAPDH,* in PANC-1 parental and *HNRNPF* SE deleted cells (n=4 biological replicates). **l** Representative immunoblot from two independent experiments showing hnRNP F levels in PANC-1 parental, *HNRNPF* SE deleted, or SE deleted cells with exogenously expressed *HNRNPF*. **m** Cell confluence determined using IncuCyte software from phase-contrast images of PANC-1 parental, *HNRNPF* SE deleted, or SE deleted cells with exogenously expressed *HNRNPF,* from 2 independent experiments. Violin plots in (**a-b**) indicate median (middle line), 25th, 75th percentile (thin dotted line). Box plots in (**i**) indicate median (middle line), 25th, 75th percentile (box), 10th and 90th percentile (whiskers), and outliers (single points). Data represent means ± SEM in (**k**), and range in (**m**). An unpaired two-tailed Mann-Whitney test was used in (**a**), Kruskal-Wallis test with Dunn's multiple comparison in (**b**), and unpaired two-tailed t-test comparing the means in (**i**) and (**k**). Source data are provided as a Source Data file.

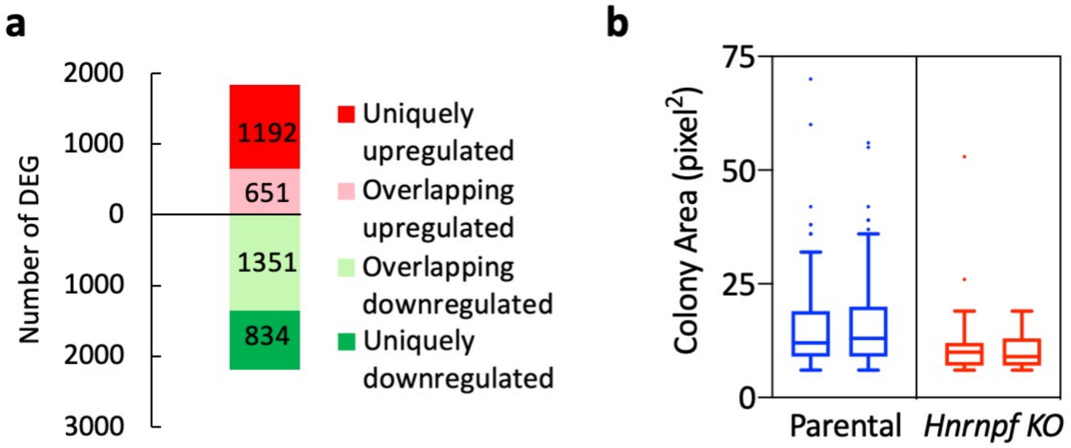

Supplementary Figure 2. ***HNRNPF*** **expression and super-enhancer in PDAC**

a Bar graph showing differentially expressed genes (DEGs) that are uniquely up- or down-regulated upon *Hnrnpf* KO compared to Parental FC1245 cells and those that overlap with DEGs in *HNRNPF* SE deleted compared to Parental Mia PaCa-2 cells. **b** Quantification of soft agar colony area from FC1245 parental and *Hnrnpf* KO cells (n=2). Box plots indicate median (middle line), 25th, 75th percentile (box), 10th and 90th percentile (whiskers), and outliers (single points). Source data are provided as a Source Data file.

Supplementary Figure 3. HNRNPF regulates PRMT1 levels

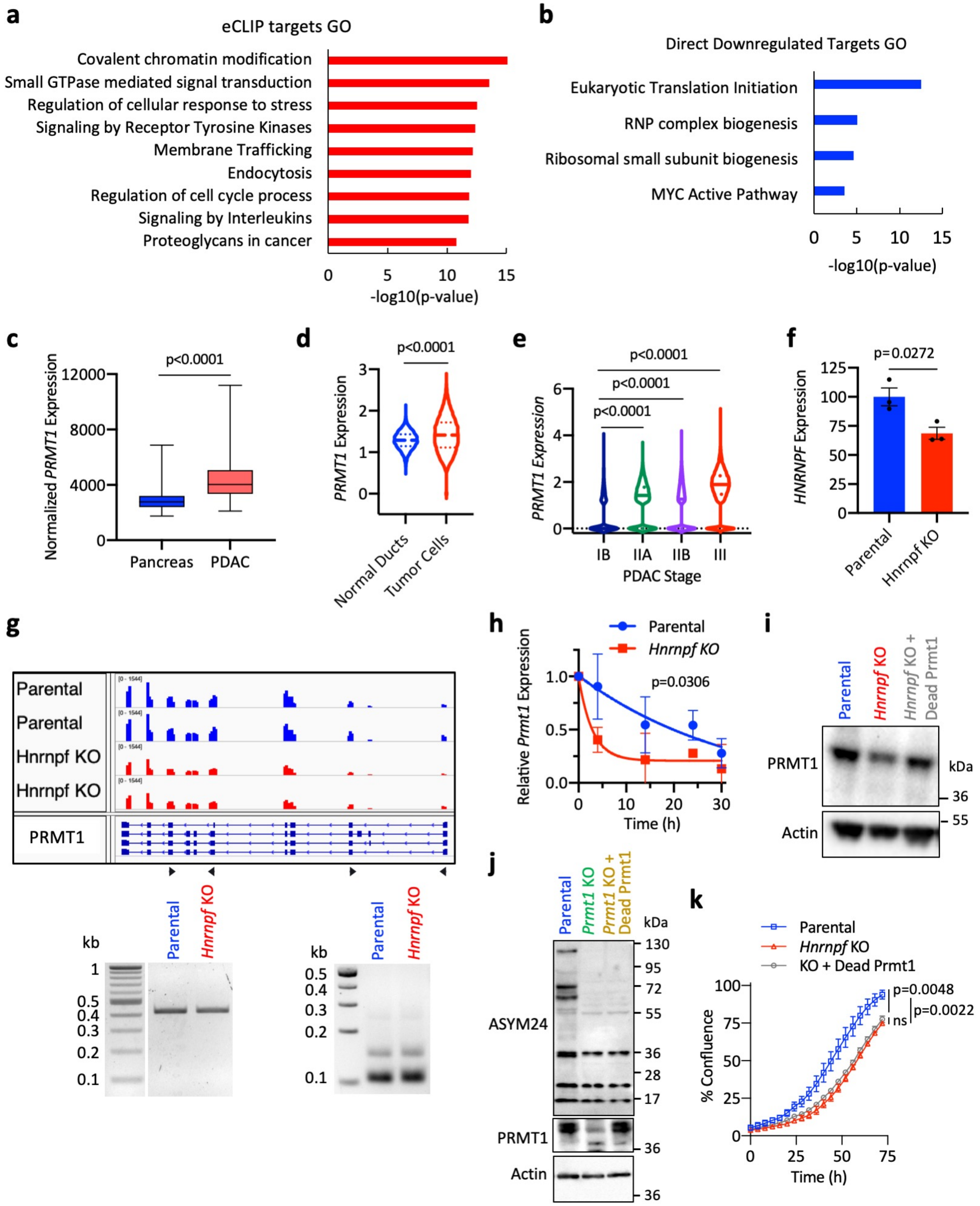

Supplementary Figure 3. HNRNPF regulates PRMT1 levels

Gene ontological analysis of (**a**) eCLIP targets in AA0779E cells and (**b)** direct eCLIP targets of hnRNP F that are downregulated upon its knockdown in AA0779E and knockout in FC1245 cells. **c** Box plot showing *PRMT1* expression in human PDAC (n=149) and normal pancreas samples (n=328), based on TCGA and GTEx data. Box plots indicate median (middle line), 25th, 75th percentile (box) and minima and maxima (whiskers). **d** Violin plot showing *PRMT1* expression derived from human single-cell RNA-seq data from normal pancreatic ducts (n=7678 cells) and malignant PDAC cells (n=13917 cells). **e** Violin plot showing *PRMT1* expression in the malignant compartment derived from human scRNA-seq data for PDAC stage IB (n=7768 cells), IIA (n=10792 cells), IIB (n=27947 cells), and III (n=17134 cells). **f** RT-qPCR showing *Prmt1* expression, normalized to *GAPDH,* in FC1245 *Hnrnpf KO* cells (n=3 biological replicates). **g** (Top) Genome browser tracks from 2 biological replicates showing mRNA reads at the *Prmt1* locus in FC1245 parental or *Hnrnpf* KO cells. (Bottom) Representative RT-PCR analysis from 2 independent experiments using primers within the exons indicated by arrows. **h** *Prmt1* mRNA decay upon transcriptional inhibition with 5µg/ml Actinomycin D in FC1245 parental and *Hnrnpf KO* cells (n=3). Data are representative of 3 independent experiments. **i** Representative immunoblot from 2 independent experiments showing Prmt1 and Actin in FC1245 parental, *Hnrnpf* KO, or *Hnrnpf* KO cells exogenously expressing catalytically dead Prmt1. **j** Representative immunoblot from 2 independent experiments showing Prmt1, Actin, and asymmetrically dimethylated arginine-containing protein levels in FC1245 parental, *Prmt1* KO, or *Prmt1* KO cells exogenously expressing catalytically dead Prmt1. **k** Cell confluence determined using IncuCyte software from phase-contrast images of FC1245 parental, *Hnrnpf* KO, or *Hnrnpf* KO cells exogenously expressing catalytically-dead PRMT1. Data are from 3 independent experiments. Violin plots in (**d-e**) indicate median (middle line), 25th, 75th percentile (thin dotted line). Data represent the mean ± SEM in (**f**) and (**h**), and mean ± SD in (**k**). An unpaired two-tailed Mann Whitney test was used in (**c**), Kruskal-Wallis test with Dunn's multiple comparison in (**e**), unpaired two-tailed t-test in (**f**), and F test in (**h**). ns: not significant. Source data are provided as a Source Data file.

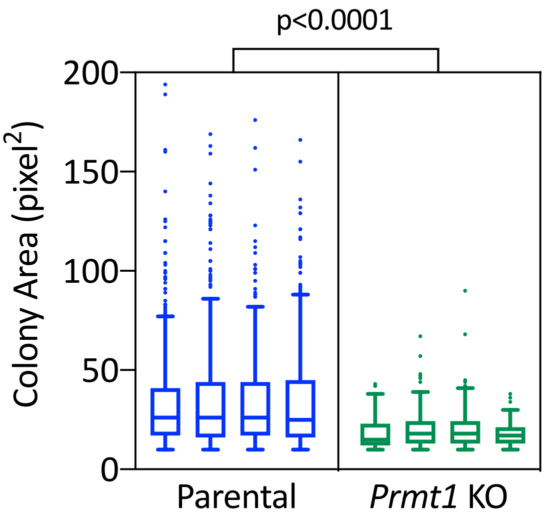

Supplementary Figure 4. Loss of *Prmt1* impedes anchorage-independent growth.
Quantification of soft agar colony area of FC1245 parental and *Prmt1l* KO cells (n=4). Box plots indicate median (middle line), 25th, 75th percentile (box), 10th and 90th percentile (whiskers), and outliers (single points). Statistics were derived from an unpaired two-tailed t-test comparing the means. Source data are provided as a Source Data file.

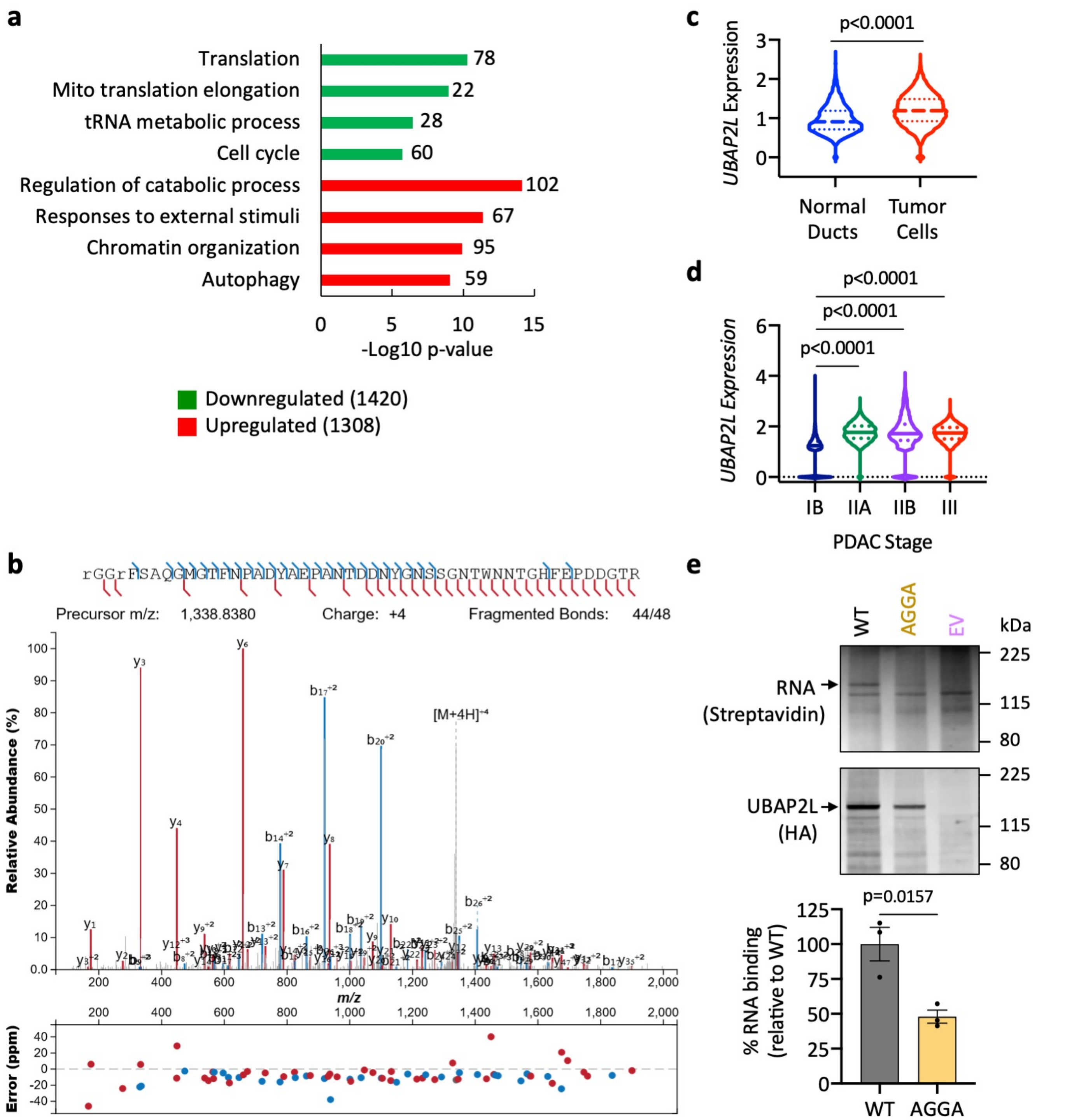

Supplementary Figure 5. PRMT1 methylates UBAP2L to regulate protein translation

a Gene ontology of up- or down-regulated genes comparing *Prmt1* KO with the parental FC1245 cells. Number of genes in each category are shown to the right of the graph. **b** MS/MS spectra of demethylated peptide from Ubap2l. Dimethylation is detected at both R's in the N terminal RGGR motif. **c** Violin plot showing *UBAP2L* expression derived from human single-cell RNA-seq data from normal pancreatic ducts (n=7678 cells) and malignant PDAC cells (n=13917 cells). **d** Violin plot showing *UBAP2L* expression in the malignant epithelial compartment derived from human single-cell RNA-seq data for PDAC stage IB (n=7768 cells), IIA (n=10792 cells), IIB (n=27947 cells), and III (n=17134 cells). (**e**) Visualization of UBAP2L-bound RNA (top blot) and UBAP2L levels (bottom blot) and quantification (bottom graph) of the relative amount of biotinylated RNA bound to WT compared to AGGA mutant HA-UBAP2L. Violin plots in (**c-d**) indicate median (middle line), 25th, 75th percentile (thin dotted line). Data represent means ± SEM from 3 biological replicates in (**e**). An unpaired two-tailed Mann Whitney test was used in (**c**), Kruskal-Wallis test with Dunn's multiple comparison in (**d**), and an unpaired two-tailed t-test in (**e**). Source data are provided as a Source Data file.

Supplementary Figure 6. ***Rpl31*** **and** ***Eef1d*** **expression in** ***Hnrnpf*** **and** ***Prmt1*** **KO cells**

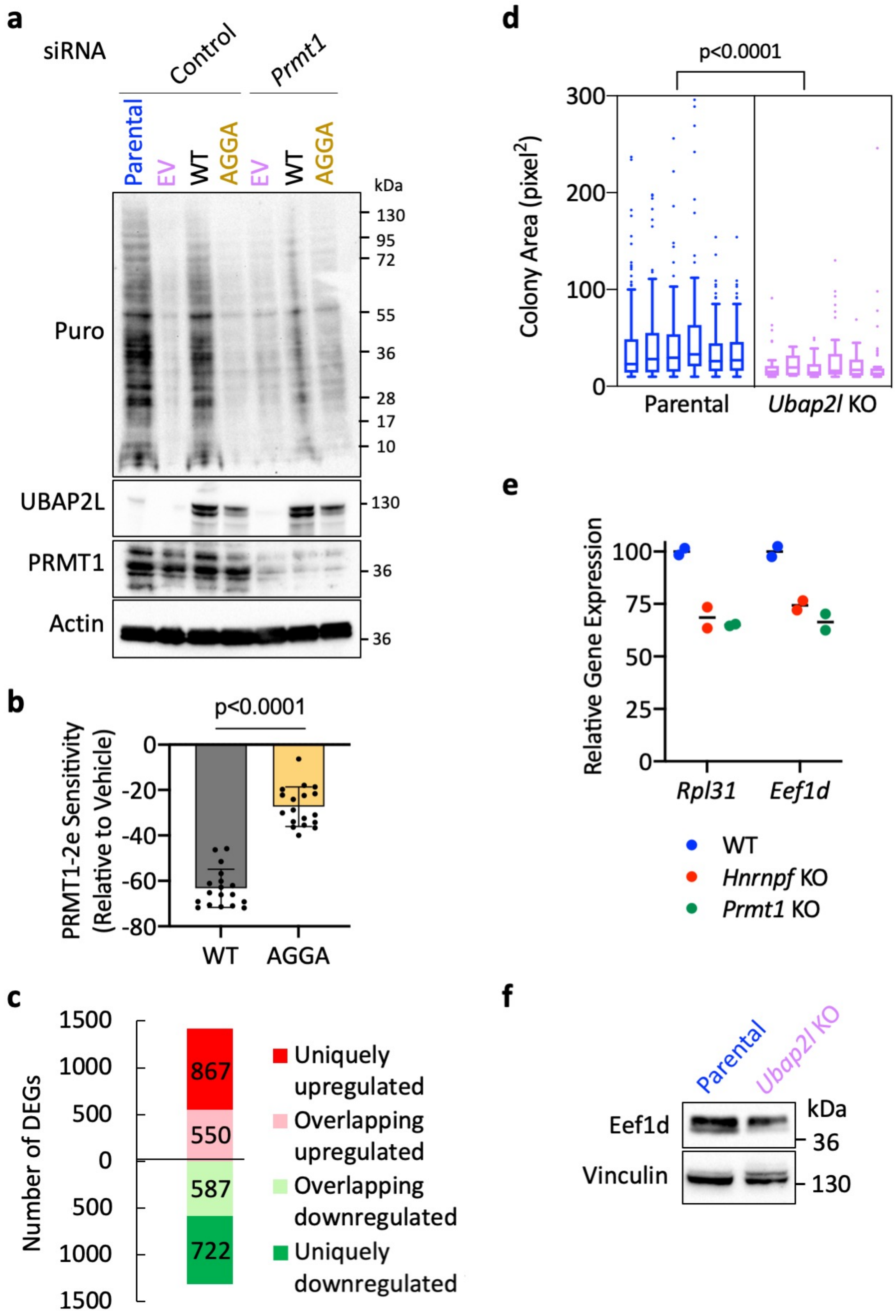

Supplementary Figure 6. *Rpl31* and *Eef1d* expression in *Hnrnpf* and *Prmt1* KO cells
a Representative immunoblot from 2 independent experiments of whole cell extracts from puromycin-treated FC1245 parental, or *Ubap2l* KO cells transiently transfected for 24 hr with either empty vector (EV), wild-type HA-tagged UBAP2L (WT) or R189A/R190A HA-UBAP2L (AGGA), two days after *Prmt1* or control knockdown. **b** Impact of PRMT1 inhibitor on cell viability of FC1245 *Ubap2l* KO cells transiently transfected with either WT or R187A/R190A Ubap2l (AGGA) after treatment with 2μM PRMT1-2e or vehicle for 2 days (n=18 biological replicates). Data are representative of 2 independent experiments. **c** Bar graph showing differentially expressed genes (DEGs) that are uniquely up- and down-regulated upon *Prmt1* KO compared to FC1245 Parental and those that overlap with DEGs in *Ubap2l* KO compared to Parental cells. **d** Quantification of soft agar colony area of FC1245 parental and *Ubap2l* KO cells (n=6). **e** Normalized mRNA expression, from RNAseq, of *Rrpl31* and *Eef1d* in *Hnrnpf* and *Prmt1* KO cells. Dots represent the 2 individual replicates and the line represents the mean. **f** Representative immunoblot of two independent experiments showing Eef1d and Vinculin levels in FC1245 Parental and *Ubap2l* KO cells. The Vinculin blot is identical to that in Figure 6e as both Eef1d and Rpl31 were probed on the same blot. Data represent mean ± SEM in (**b**). Box plots in (**d)** indicate median (middle line), 25th, 75th percentile (box), 10th and 90th percentile (whiskers), and outliers (single points). Unpaired two-tailed t-tests comparing the means were used in (**b**) and (**d**). Source data are provided as a Source Data file.

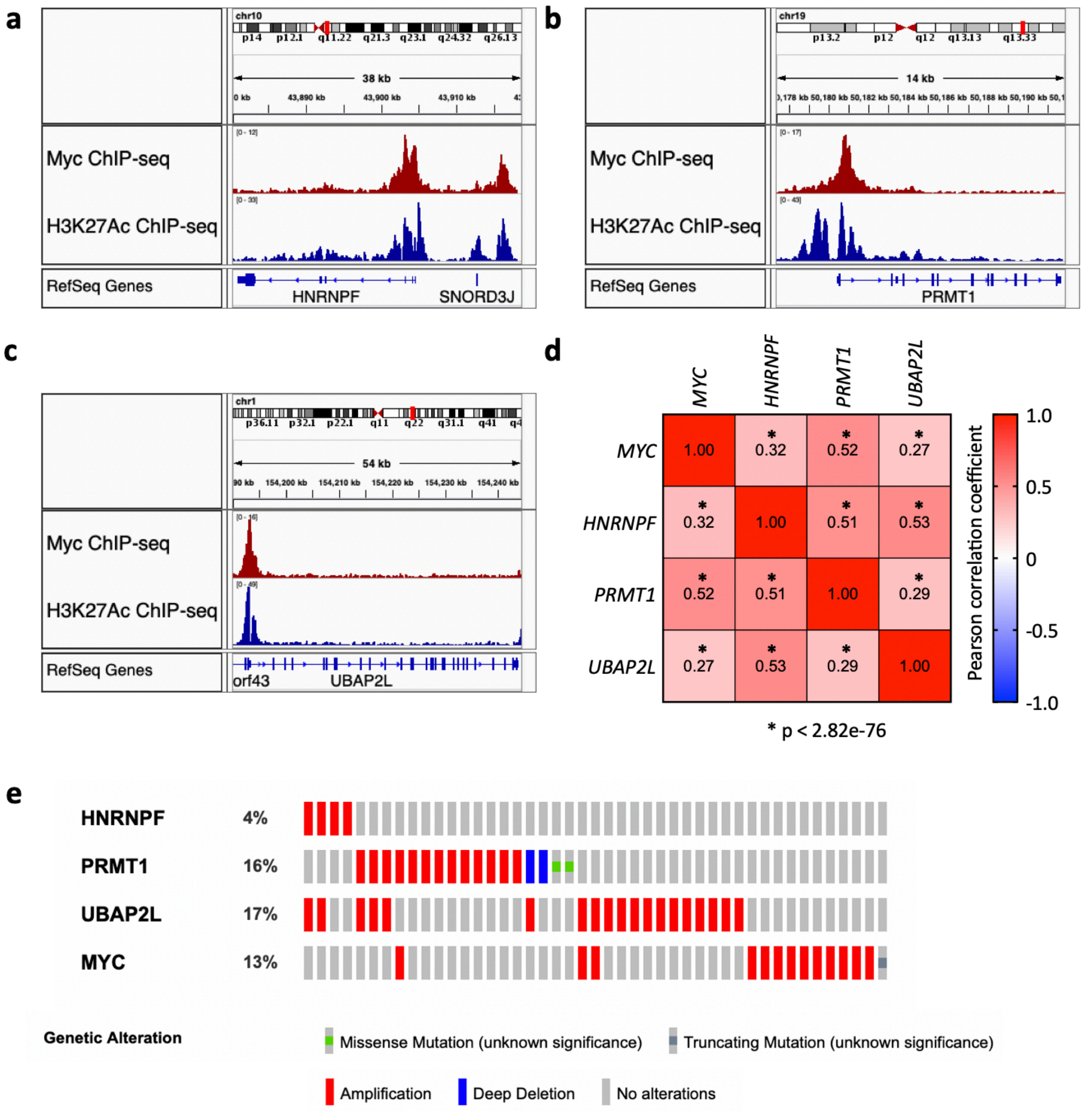

Supplementary Figure 7. Myc binds to the HNRNPF, PRMT1, and UBAP2L loci

Genome browser tracks showing Myc binding at the (**a**) *HNRNPF* promoter and enhancer, and (**b**) *PRMT1* and (**c**) *UBAP2L* promoters in MIA PaCa-2 cells (GEO accession GSE143804). **d** Two-tailed Pearson correlation, on a cell by cell basis, between *MYC*, *HNRNPF*, *PRMT1*, and *UBAP2L* derived from their expression in tumor cells from human PDAC scRNA-seq data. **e** OncoPrint diagram of mutational frequencies and types of alterations of the indicated genes in 109 laser-capture microdissected human PDAC samples. Source data are provided as a Source Data file.

Supplementary Figure 8. Myc expression sensitizes to PRMT1 inhibition

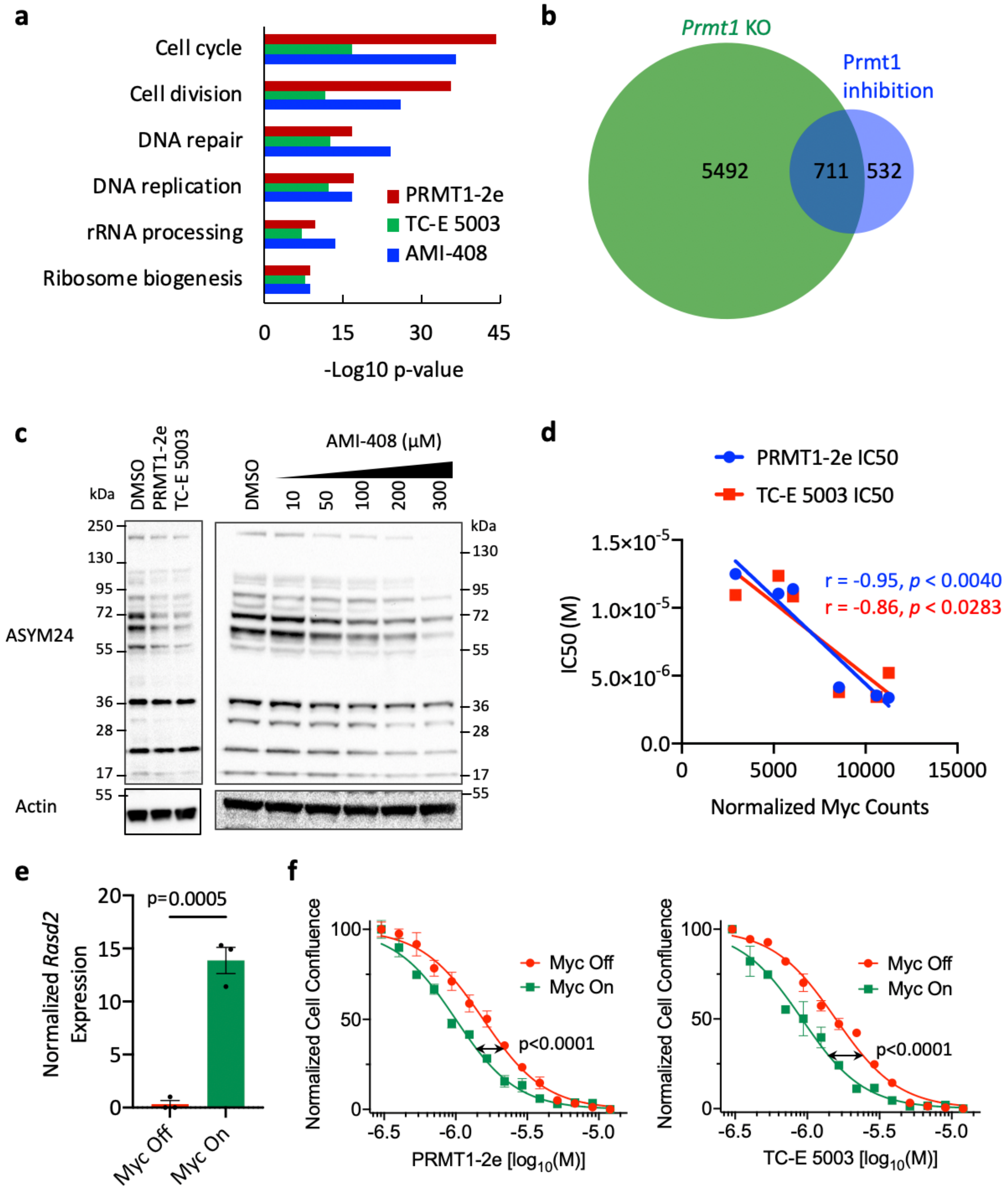

Supplementary Figure 8. Myc expression sensitizes to PRMT1 inhibition

a Gene ontological analysis of differentially expressed genes from FC1245 cells treated with the PRMT1 inhibitors listed. **b** Venn diagram showing the overlap in differentially expressed genes common to the PRMT1 inhibitors PRMT1-2e/TC-E 5003 and AMI-408 and *Prmt1* knockout cells. **c** Representative immunoblot from 2 independent experiments showing asymmetrically dimethylated arginine-containing protein levels and Actin in FC1245 cells treated with PRMT1-2e or TC-E 5003 (left) and immunoblot from 1 experiment in which cells were treated with increasing concentration of AMI-408. **d** Two-tailed Person correlation between normalized *Myc* counts and PRMT1 inhibitors PRMT1-2e and TC-E 5003 (n=6 organoid lines). **e** RT-qPCR showing *Rasd2* expression, normalized to *Actb,* in FB21.3F cells (n=3 biological replicates). **f** Dose-response curves for the indicated PRMT1 inhibitors in FB21.3F cells seeded in the presence of 4-OHT (Myc On) or vehicle (Myc Off). n=3 biological replicates. Data are representative of 3 independent experiments with similar results. Data represent the mean ± SEM in (**e-f**). Statistics were derived from an unpaired two-tailed t-test in (**e**) and an F test comparing Dose-Response EC50 shift in (**f**). Source data are provided as a Source Data file.

UNCROPPED WESTERN BLOTS

Supplementary Figure 1l

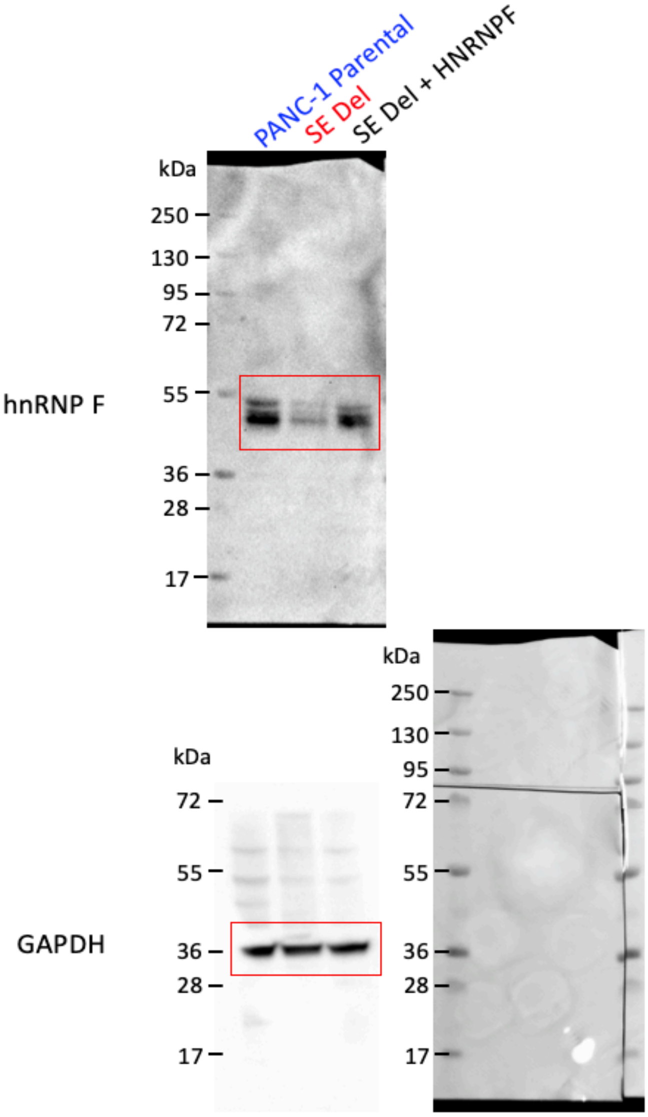

Supplementary Figure 3i

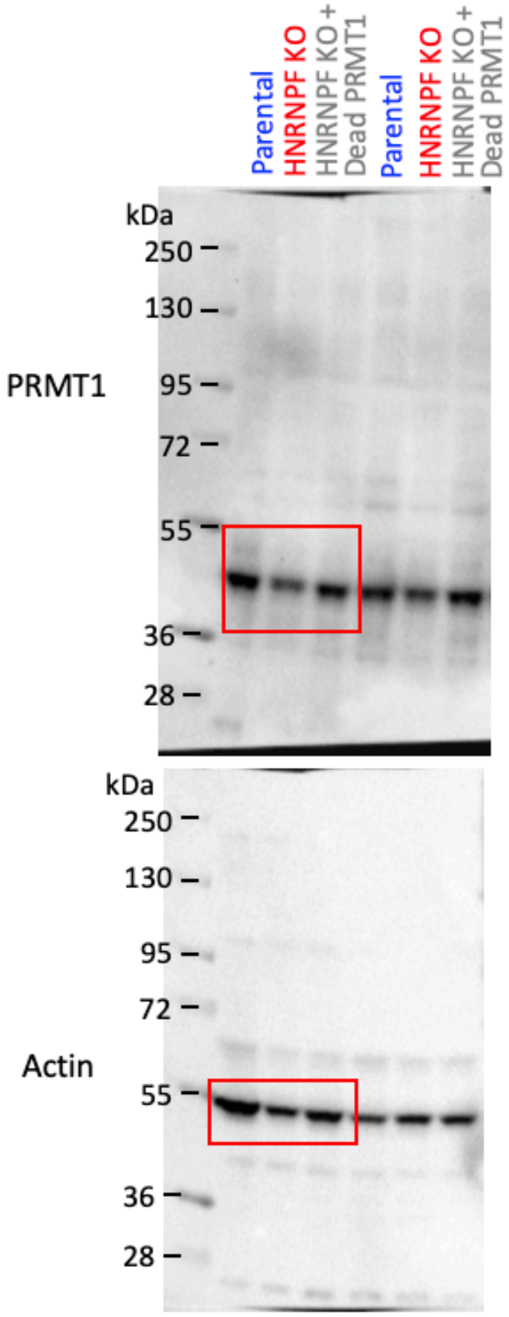

Supplementary Figure 3j

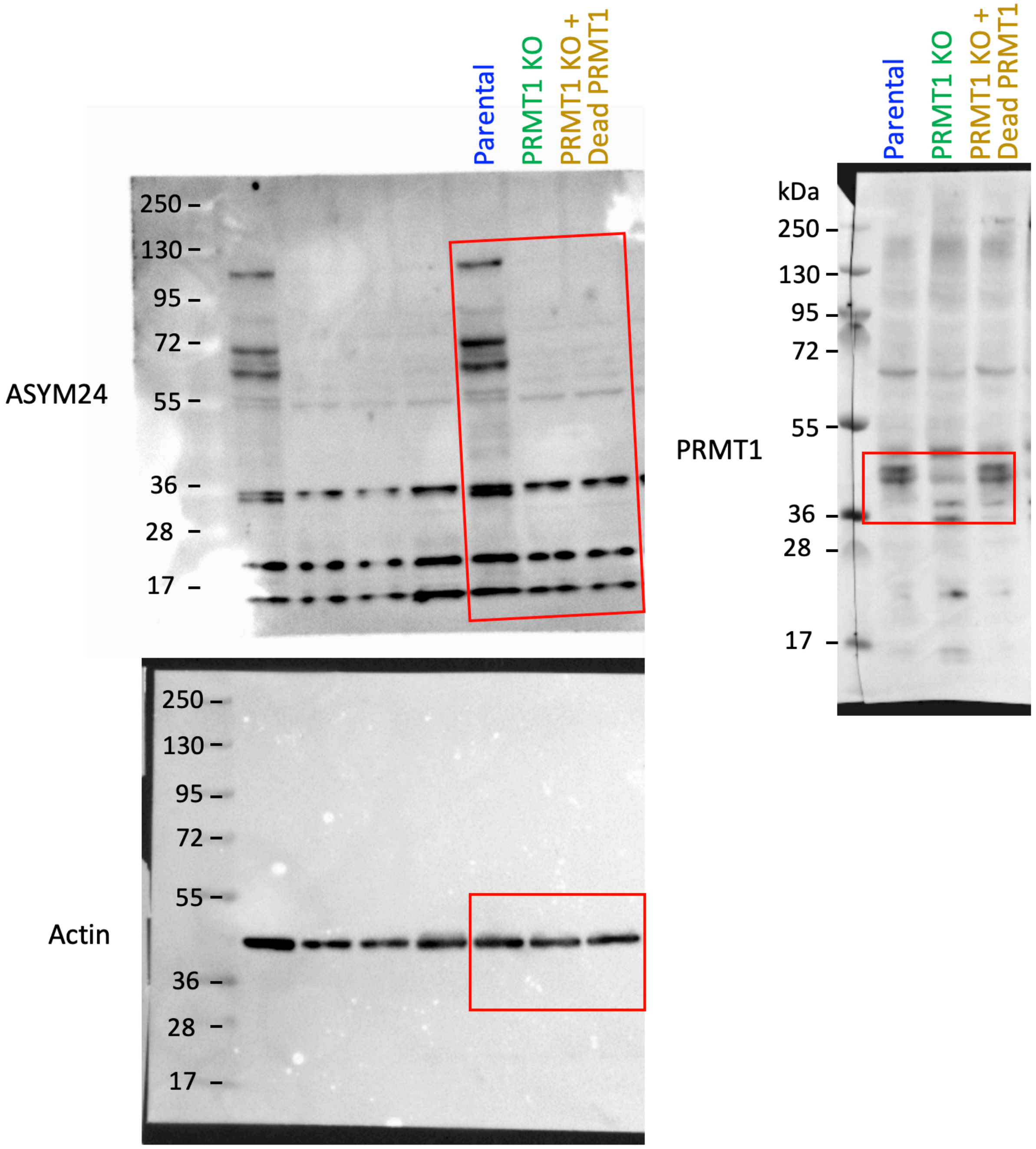

Supplementary Figure 5e

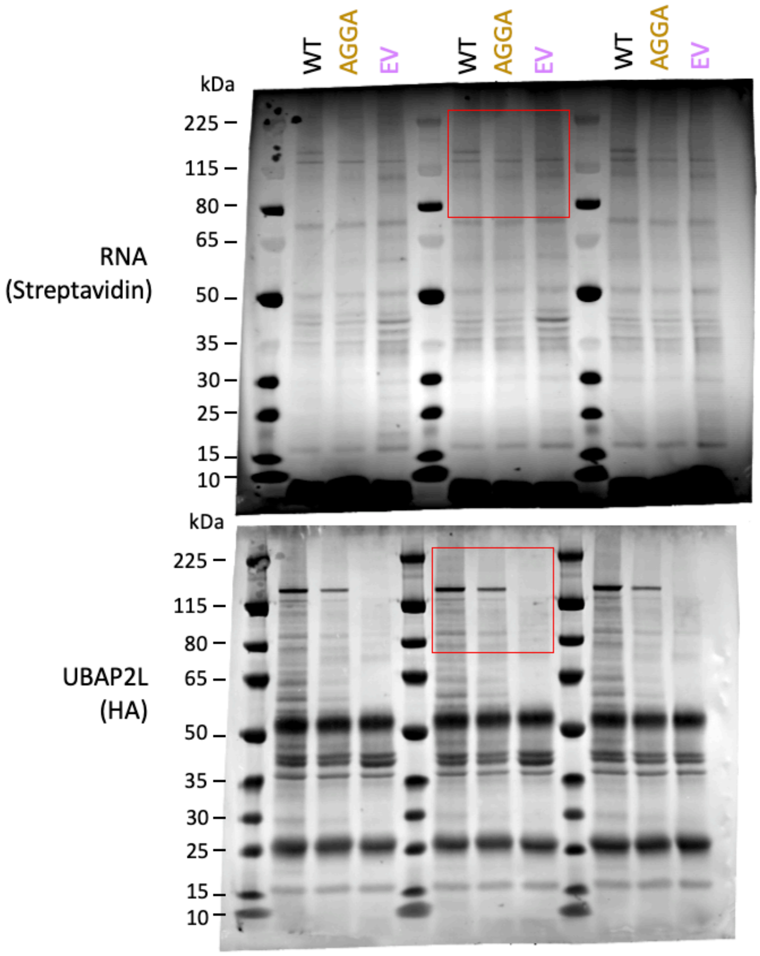

Supplementary Figure 6a

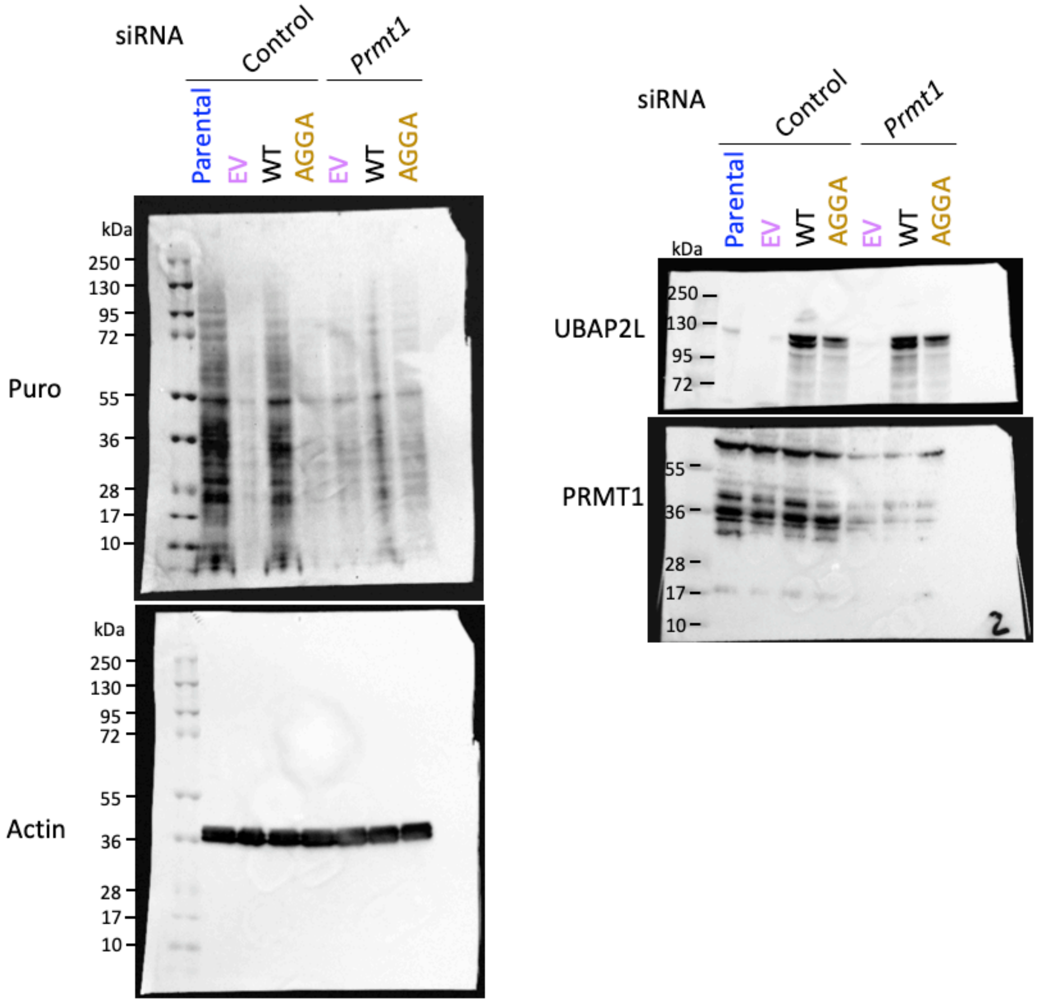

Supplementary Figure 6f

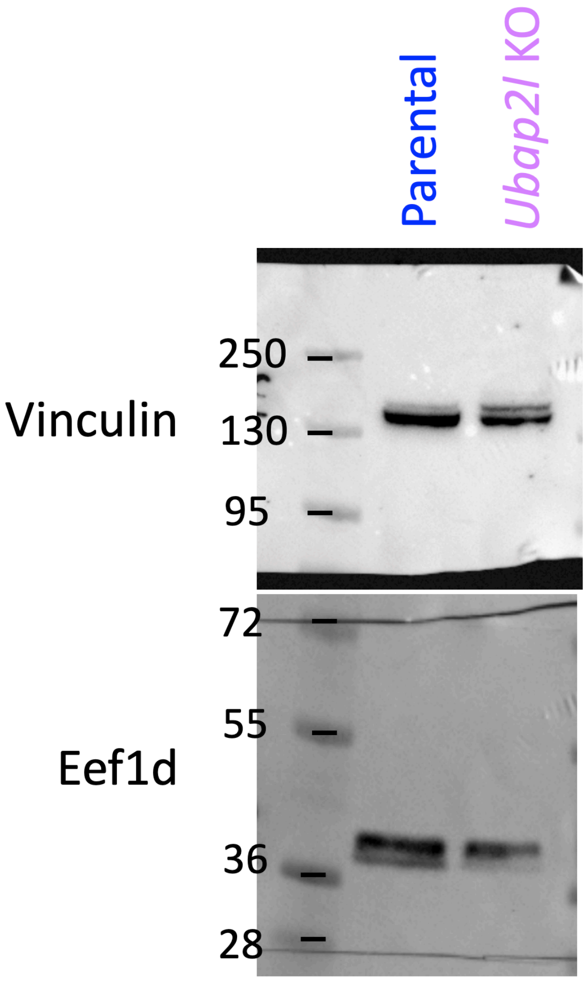

Supplementary Figure 8c

Red boxes denote areas that were included in the figure
Ublabeled samples were treated with lower drug concentrations and were not included in this manuscript

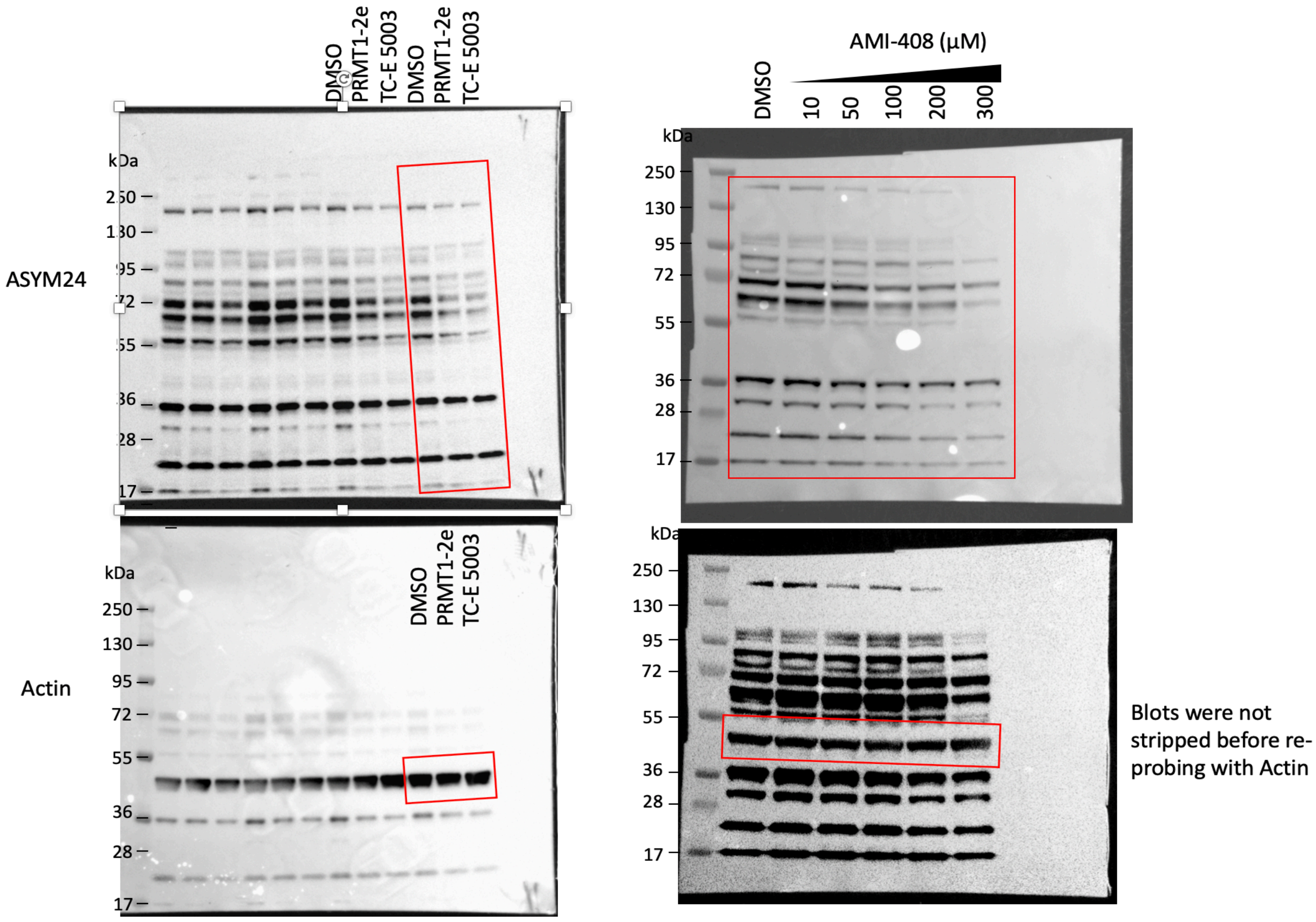

Blots were not stripped before re-probing with Actin
