# Supplementary material for: A super-enhancer-regulated RNA-binding protein cascade drives pancreatic cancer
Source: Nat Commun. 2023 Sep 6;14:5195. doi: 10.1038/s41467-023-40798-6 (PMC10482938; doi:10.1038/s41467-023-40798-6)
Supplement: Supplementary file 2 — Description of Additional Supplementary Files [file 41467_2023_40798_MOESM2_ESM.pdf]

Description of Additional Supplementary Files

Supplementary Data 1. hnRNP F eCLIP targets

List of all hnRNP F bound RNAs as identified by eCLIP.

Supplementary Data 2. Changes in asymmetric dimethyl arginines induced by loss of Prmt1

Asymmetric dimethyl arginine peptides identified by quantitative mass spectroscopy in FC1245 Parental and *Prmt1* KO cells.

Supplementary Data 3. ChIP-seq and ATAC-seq quality control

Table displaying read counts for ChIP-seq and ATAC-seq data.

Supplementary Data 4. RT-qPCR, sgRNA, and PCR primer sequences

RT-qPCR, sgRNA, and PCR primer sequences utilized in this manuscript. “h” denotes human origin, and “m” denotes mouse origin.
