# Supplementary material for: A super-enhancer-regulated RNA-binding protein cascade drives pancreatic cancer
Source: Nat Commun. 2023 Sep 6;14:5195. doi: 10.1038/s41467-023-40798-6 (PMC10482938; doi:10.1038/s41467-023-40798-6)
Supplement: Supplementary file 7 — Reporting Summary [file 41467_2023_40798_MOESM7_ESM.pdf]

nature portfolio

Corresponding author(s): Ronald M. Evans and Michael Downes

Last updated by author(s): Jul 7, 2023

Reporting Summary

Nature Portfolio wishes to improve the reproducibility of the work that we publish. This form provides structure for consistency and transparency in reporting. For further information on Nature Portfolio policies, see our Editorial Policies and the Editorial Policy Checklist.

Statistics

For all statistical analyses, confirm that the following items are present in the figure legend, table legend, main text, or Methods section.

n/a	Confirmed	
☐	☒	The exact sample size (*n*) for each experimental group/condition, given as a discrete number and unit of measurement
☐	☒	A statement on whether measurements were taken from distinct samples or whether the same sample was measured repeatedly
☐	☒	The statistical test(s) used AND whether they are one- or two-sided<br>*Only common tests should be described solely by name; describe more complex techniques in the Methods section.*
☒	☐	A description of all covariates tested
☐	☒	A description of any assumptions or corrections, such as tests of normality and adjustment for multiple comparisons
☐	☒	A full description of the statistical parameters including central tendency (e.g. means) or other basic estimates (e.g. regression coefficient) AND variation (e.g. standard deviation) or associated estimates of uncertainty (e.g. confidence intervals)
☐	☒	For null hypothesis testing, the test statistic (e.g. *F*, *t*, *r*) with confidence intervals, effect sizes, degrees of freedom and *P* value noted<br>*Give P values as exact values whenever suitable.*
☒	☐	For Bayesian analysis, information on the choice of priors and Markov chain Monte Carlo settings
☒	☐	For hierarchical and complex designs, identification of the appropriate level for tests and full reporting of outcomes
☐	☒	Estimates of effect sizes (e.g. Cohen's *d*, Pearson's *r*), indicating how they were calculated

Our web collection on statistics for biologists contains articles on many of the points above.

Software and code

Policy information about availability of computer code

Data collection	Cell proliferation assays and Caspase-3/7 assays were performed on the IncuCyte S3 v2019A system (Essen BioScience).<br>RT-qPCR was performed on the Bio-Rad CFX384 detection system using Bio-Rad CFX Maestro software v2.3.<br>Copy-number analysis was performed using Agilent 2565C DNA scanner.<br>Images were acquired on the Revolve (Echo Laboratories) and Olympus BX43 microscopes.
Data analysis	All code and analyses used in the present study are publicly available.<br>For eCLIP, the current processing pipeline can be found at https://github.com/YeoLab/eclip.<br>The RNA-seq data analysis was performed in R v4.0.2 and web-based programs with the following packages: STAR v2.7.1a, Cuffdiff v2.2.1, DESeq2 v1.30.1, TopHat2 v2.0.4, DAVID v6.8.<br>For splicing detection, the vast-tools (v 2.0.2) were used.<br>For ChIP-seq and ATAC-seq, data analysis was performed in R with the following packages: Bowtie2 and Homer v4.11.1.<br>sgRNAs were designed using E-CRISP v5.4 and CHOPCHOP v3.<br>Copy number analysis data were analyzed with Agilent Feature Extraction v11.0.<br>Western blot band intensities were quantified with Image Lab 5.2.1, ImageJ 2.0, and Fiji v2.0.0-rc-43/1.52n.<br>The tissue microarray images were analyzed using Aperio ImageScope (v12.4.6.5003).<br>All statistical tests were performed using Prism software version 9.0 for mac OS X (GraphPad Software).<br>ChIP-seq and ATAC-seq data tracks were visualized using IGV v2.3.90.

For manuscripts utilizing custom algorithms or software that are central to the research but not yet described in published literature, software must be made available to editors and reviewers. We strongly encourage code deposition in a community repository (e.g. GitHub). See the Nature Portfolio guidelines for submitting code & software for further information.

Data

Policy information about availability of data

All manuscripts must include a data availability statement. This statement should provide the following information, where applicable:

- Accession codes, unique identifiers, or web links for publicly available datasets
- A description of any restrictions on data availability
- For clinical datasets or third party data, please ensure that the statement adheres to our policy

The publicly available scRNA-seq data sets used in this study are available in the National Genomics Data Center under accession code PRJCA00106330 and the Gene Expression Omnibus (GEO) database under accession codes GSE20205188 and GSE15477850. The publicly available Myc ChIP-seq data used in this study are available under accession codes GSE14380449. Normal pancreas H3K27Ac ChIP-seq data were retrieved from GSM1013129 and GSM906397. Normal cell H3K27Ac ChIP-seq data from 293T, NHEK, myoblasts, monocytes, bronchial epithelial cells, skeletal myotubes, keratinocytes, and macrophages were downloaded from GSM217141696, GSM1666386, GSM4143867, GSM3462802, GSM3892733, GSM3611923, GSM1645725, and GSM1327358, respectively. H3K27Ac ChIP-seq data from cancer cell lines K562, MCF7, He-La-S3, HepG2, Dnd41, A549, and HCT-116 H3K27Ac ChIP-seq were retrieved from the ENCODE database97 (GSM733656, GSM945854, GSM733684, GSM733743, GSM1003462, GSM1003578, and GSM945853, respectively). PANC-1 BRD4 ChIP-seq data were retrieved from PRJEB2786398. The RNA-seq, ChIP-seq, ATAC-seq and eCLIP data reported in this paper have been deposited in the National Center for Biotechnology Information (NCBI) Sequence Read Archive (SRA) database, under accession codes PRJNA678286 and GSE234078. The mass spectrometry proteomics data are deposited in the ProteomeXchange Consortium via the PRIDE partner repository with the dataset identifier PXD030423. The remaining data are available within the Article, Supplementary Information, Supplementary Data, or Source Data file.

Research involving human participants, their data, or biological material

Policy information about studies with human participants or human data. See also policy information about sex, gender (identity/presentation), and sexual orientation and race, ethnicity and racism.

Reporting on sex and gender	Only one biopsy from a self-reported male was successfully obtained, analyzed, and reported in this manuscript, so no sex-based analysis was performed. The patient gave written informed consent.
Reporting on race, ethnicity, or other socially relevant groupings	This information was not available to the researchers analyzing the biopsy.
Population characteristics	64-year old male
Recruitment	Patients were selected based upon their eligibility for the protocol (clinicaltrials.gov NCT03117920). Inclusion Criteria:<br>Willing and able to provide written informed consent.<br>Ability to comply with the protocol.<br>Aged ≥ 18 years.<br>Histologically or cytologically confirmed metastatic pancreatic adenocarcinoma that has progressed on one or more chemotherapy regimens.<br>Karnofsky performance status ≥ 70%.<br>At least one lesion that can be measured accurately at baseline as ≥10mm in the longest diameter (except lymph nodes which must have a short axis ≥15mm) with CT/MRI and which is suitable for repeated measurements per RECIST v1.1<br>Adequate haematological and end-organ function, as per the local institutions reference ranges, within 72 hrs prior to day 1 of cycle 1 of treatment defined by the following:<br>Life expectancy ≥ 12 weeks.<br>Negative pregnancy test within 14 days of day 1 cycle 1 for female patients of childbearing potential.<br>Tumour sites amenable to repeated biopsies.<br>Willingness to undergo paired tumour biopsies during the trial.<br>Agreement to use adequate contraception from 2 weeks before the start of treatment with Minnelide and until 90 days after completion of treatment.
Ethics oversight	WIRB 20170433 and IRB 18-0005

Note that full information on the approval of the study protocol must also be provided in the manuscript.

Field-specific reporting

Please select the one below that is the best fit for your research. If you are not sure, read the appropriate sections before making your selection.

- [x] Life sciences
- [ ] Behavioural & social sciences
- [ ] Ecological, evolutionary & environmental sciences

For a reference copy of the document with all sections, see nature.com/documents/nr-reporting-summary-flat.pdf

Life sciences study design

All studies must disclose on these points even when the disclosure is negative.

Sample size	A minimal number of animals for statically significant results were used in compliance with IACUC guidelines. For in vitro experiments, sample

	size was based on the variability of the data, the purpose of the experiment, the available resources, and previous data from similar experiments, in order to maintain the balance between reaching statistical significance and minimizing the number of animal/reagents used.
Data exclusions	For IHC on tissue arrays, biopsies that no longer contained tumor cells due to sectioning beyond the margins of the tumor were excluded based on pre-established exclusion criteria.
Replication	Each experiment was repeated at least twice as stated in the figure legends. All attempts at replication were successful.
Randomization	Animals were randomly assigned to treatment or orthotopic transplant groups. For in vitro experiments, randomization was not relevant as the samples were treated and analyzed in the same manner.
Blinding	Investigators were blinded for the tumor measurements. Investigators were not blinded during outcome assessment for other experiments since data acquisition and analysis were done using the indicated software.

Reporting for specific materials, systems and methods

We require information from authors about some types of materials, experimental systems and methods used in many studies. Here, indicate whether each material, system or method listed is relevant to your study. If you are not sure if a list item applies to your research, read the appropriate section before selecting a response.

Materials & experimental systems

n/a	Involved in the study
☐	☒ Antibodies
☐	☒ Eukaryotic cell lines
☒	☐ Palaeontology and archaeology
☐	☒ Animals and other organisms
☒	☐ Clinical data
☒	☐ Dual use research of concern
☒	☐ Plants

Methods

n/a	Involved in the study
☐	☒ ChIP-seq
☒	☐ Flow cytometry
☒	☐ MRI-based neuroimaging

Antibodies

Antibodies used	H3K27Ac Abcam Cat# ab4729, RRID: AB_2118291, 1µg<br>c-Myc Cell Signaling Technology Cat# 9402, RRID:AB_2151827, 1:50<br>ERalpha Santa Cruz Biotechnology Cat# sc-543, RRID:AB_631471, 1µg<br>human hnRNP F Santa Cruz Biotechnology Cat# sc-32309, RRID:AB_627732, 1:1000<br>mouse/human hnRNP F Abcam Cat# ab50982, RRID:AB_880477, 1:1000<br>PRMT1 R and D Systems Cat# AF6016, RRID:AB_1964684, 1:1000<br>Vinculin R and D Systems Cat# MAB6896, RRID:AB_10992930, 1:1000<br>human UBAP2L Bethyl Cat# A300-533A, RRID:AB_477953, 1:10000<br>mouse/human UBAP2L Bethyl Cat# A300-534A, RRID:AB_2272582, 1:5000<br>human UBAP2L Sigma-Aldrich Cat# HPA035068, RRID:AB_10696366, 1:500 for IHC<br>Anti-dimethyl-Arginine Antibody, asymmetric Millipore at# 07-414, RRID:AB_310596, 1:1000<br>Puromycin Millipore Cat# MABE343, RRID:AB_2566826, 1:10,000<br>B-Actin D6A8 Cell Signaling Technology Cat# 8457, RRID:AB_10950489, 1:1000<br>RPL31 ABclonal Cat# A17527, RRID:AB_2772081, 1:1000 for WB and 1:200 for IHC<br>EEF1D ABclonal Cat# A2509, RRID:AB_2764400, 1:1000<br>Ki67 GeneTex Cat# GTX16667, RRID:AB_422351, 1:50<br>anti-rabbit IgG-HRP Santa Cruz Biotechnology Cat# sc-2004, RRID:AB_631746, 1:10,000<br>anti-mouse IgG2a -HRP Thermo Fisher Scientific Cat# M32207, RRID:AB_2536640, 1:10,000<br>anti-mouse IgG-HRP Santa Cruz Biotechnology Cat# sc-2005, RRID:AB_631736, 1:10,000<br>anti-goat IgG-HRP Millipore Cat# 401515-2ML, RRID:AB_10682600, 1:10,000<br>anti-HA antibody Abcam Cat# ab49969, RRID:AB_880330, 1 µg/ml<br>IRDye 800CW goat anti-mouse IgG secondary antibody LI-COR Biosciences Cat# 926-32210, RRID:AB_621842, 0.1 µg/ml<br>hnRNP F Mybiosource Cat# MBS178697, 1:1000
Validation	Pre-validated commercially available antibodies were used. All antibodies have been tested by the manufacturer for the relevant application on cells expressing the antigen. hnRNP F Santa Cruz Biotechnology Cat# sc-32309 was validated for detection of hnRNP F of human origin by WB. anti-HA antibody Abcam Cat# ab49969 and Puromycin Millipore Cat# MABE343 are suitable for WB and are species independent. H3K27Ac Abcam Cat# ab4729 is suitable for ChIP and reacts with human. c-Myc Cell Signaling Technology Cat# 9402 is validated for ChIP using SimpleChIP® Enzymatic Chromatin IP Kits and reacts with mouse. PRMT1 R and D Systems Cat# AF6016, Vinculin R and D Systems Cat# MAB6896, hnRNP F Abcam Cat# ab50982, b-Actin D6A8 Cell Signaling Technology Cat# 8457, and anti-dimethyl-Arginine Antibody, asymmetric Millipore Cat# 07-414 are suitable for WB and react with mouse and human. UBAP2L Bethyl Cat# A300-533A is suitable for WB and reacts with human. UBAP2L Bethyl Cat# A300-534A is suitable for WB and IP and reacts with mouse and human. UBAP2L Sigma-Aldrich Cat# HPA035068 is suitable for IHC and reacts with human. RPL31 ABclonal Cat# A17527 and Ki67 GeneTex Cat# GTX16667 are suitable for WB and IHC-P and react with mouse. EEF1D ABclonal Cat# A2509 is suitable for WB and reacts with mouse. IRDye 800CW goat anti-mouse IgG secondary antibody LI-COR Biosciences Cat# 926-32210

and anti-mouse IgG-HRP Santa Cruz Biotechnology Cat# sc-2005 are suitable for WB and react with mouse IgG. anti-goat IgG-HRP Millipore Cat# 401515-2ML is suitable for WB reacts with goat IgG. anti-mouse IgG2a-HRP Thermo Fisher Scientific Cat# M32207 is suitable for WB and reacts with mouse IgG2a. anti-rabbit IgG-HRP Santa Cruz Biotechnology Cat# sc-2004 is suitable for WB and reacts with rabbit IgG. hnRNP F Mybiosource Cat# MBS178697 is suitable for IHC-P and reacts with human. We have previously validated ERα (Santa Cruz Biotechnology Cat# sc-543 for ChIP in the same mouse cell lines. We independently valdidated the hnRNP F, PRMT1 and UBAP2L antibodies in knockout and over-expression systems.

Eukaryotic cell lines

Policy information about cell lines and Sex and Gender in Research

Cell line source(s)	MIA PaCa-2 (CMR-CRL-1420), PANC-1 (CRL-1469), Capan-1 (HTB-79), Capan-2 (HTB-80), Hs766T (HTB-134), PSN1 (CRM-CRL-3211), Panc02.03 (CRL-2553), Panc03.27 (CRL-2549), and Su.86.86 (CRL-1837) were acquired from ATCC and YAPC (ACC 382), HupT4 (ACC 223), KCI-MOH1 (ACC 498), PaTu8902 (ACC 179), and PaTu8988T (ACC 162) were from DSMZ. FC1245 and organoid lines hT3, hF3, hF23, hF44, hM1E, and hM19A were from Dr. David Tuveson (CSHL). MUTJ cells were obtained from the University of Arizona Cancer Center. AA0779E cells were from Andrew Lowy (UCSD). DF3.4F48 and FB21.3F were from Gerard Evan (Crick).
Authentication	Cell lines were obtained directly from ATCC and DSMZ and maintained frozen until use. None of the cells lines were further authenticated.
Mycoplasma contamination	All cell lines were routinely tested for mycoplasma and tested negative.
Commonly misidentified lines (See ICLAC register)	KCI-MOH1 is a derivate of the human PDAC cell line HPAC, and as such, we included it in our analysis of human PDAC cell lines without authentication.

Animals and other research organisms

Policy information about studies involving animals; ARRIVE guidelines recommended for reporting animal research, and Sex and Gender in Research

Laboratory animals	8 to10 week old age-matched male C57BL/6J (Jackson Laboratory Cat #000664, RRID:IMSR_JAX:000664) and 9 week old male NCG (Charles River Laboratories NOD-Prkdcem26Cd52Il2rgem26Cd22/NjuCrl, RRID:IMSR_CRL:572) mice were used.
Wild animals	The study does not involve wild animals.
Reporting on sex	Only male mice were used for the study to avoid hormonal fluctuations that might impact tumor growth in female mice.
Field-collected samples	The study did not involve field-collected samples.
Ethics oversight	All procedures involving animals were performed in accordance with protocols approved by the IACUC and Animal Resources Department of the Salk Institute for Biological Studies (protocol # 11-00032).

Note that full information on the approval of the study protocol must also be provided in the manuscript.

Plants

Seed stocks	*Report on the source of all seed stocks or other plant material used. If applicable, state the seed stock centre and catalogue number. If plant specimens were collected from the field, describe the collection location, date and sampling procedures.*
Novel plant genotypes	*Describe the methods by which all novel plant genotypes were produced. This includes those generated by transgenic approaches, gene editing, chemical/radiation-based mutagenesis and hybridization. For transgenic lines, describe the transformation method, the number of independent lines analyzed and the generation upon which experiments were performed. For gene-edited lines, describe the editor used, the endogenous sequence targeted for editing, the targeting guide RNA sequence (if applicable) and how the editor was applied.*
Authentication	*Describe any authentication procedures for each seed stock used or novel genotype generated. Describe any experiments used to assess the effect of a mutation and, where applicable, how potential secondary effects (e.g. second site T-DNA insertions, mosiacism, off-target gene editing) were examined.*

ChIP-seq

Data deposition

- [x] Confirm that both raw and final processed data have been deposited in a public database such as GEO.
- [x] Confirm that you have deposited or provided access to graph files (e.g. BED files) for the called peaks.

Data access links<br>*May remain private before publication.*	https://www.ncbi.nlm.nih.gov/bioproject/PRJNA678286<br>https://www.ncbi.nlm.nih.gov/geo/query/acc.cgi?acc=GSE234078
Files in database submission	YAPC_input<br>YAPC_H3K27Ac

Mutj_input
Mutj_H3K27Ac
CAPAN1_input
CAPAN1_H3K27Ac
CAPAN2_input
CAPAN2_H3K27Ac
PaTu8988T_input
PaTu8988T_H3K27Ac
PaTu8902_input
PaTu8902_H3K27Ac
KCIMOH1_input
KCIMOH1_H3K27Ac
HupT4_Input
HupT4_H3K27Ac
HS766T_input
HS766T_H3K27Ac
Panc1_Input
Panc1_H3K27Ac
Panc02_03_Input
Panc02_03_H3K27Ac
Panc03_27_Input
Panc03_27_H3K27Ac
MiaPaca2_Input
MiaPaca2_H3K27Ac
AA0779E_Input
AA0779E_H3K27Ac
SU86_86_Input
SU86_86_H3K27Ac
PSN1_Input
PSN1_H3K27Ac
ON_24h_4O_M
ON_24h_4O_E
ON_24h_4O_In
ON_24h_Et_M
ON_24h_Et_E
ON_24h_Et_In
ON_6h_4O_M
ON_6h_4O_E
ON_6h_4O_In
ON_6h_Et_M
ON_6h_Et_E
ON_6h_Et_In

Genome browser session (e.g. UCSC): no longer applicable

Methodology

Replicates: No techincal replicates were done as each cell line represents a biological replicate.

Sequencing depth: single-end 100 bp length

Antibodies:
H3K27Ac (Abcam Cat# ab4729, RRID: AB_2118291).
c-Myc (Cell Signaling Technology Cat# 9402, RRID:AB_2151827)
ERα (Santa Cruz Biotechnology Cat# sc-543, RRID:AB_631471)

Peak calling parameters: Mouse and human fastq files were mapped using Bowtie2 to the MGSCv37 (mm9) or GRCh37 (hg19) genomes, respectively. Differential peaks were called using HOMER's default settings (Fold change >4, p-value <0.0001) using '-style factor'.

Data quality: All peaks had a FDR <5% and were >4-fold over background.

Software: Data analysis was performed in R with the following packages: Bowtie2 and Homer v4.11.1.
